# Immunosuppressive and antiinfectious regimens in vascular composite allograft recipients—a systematic review

**DOI:** 10.3389/frtra.2025.1714886

**Published:** 2025-12-18

**Authors:** Leonard Knoedler, Tobias Niederegger, Thomas Schaschinger, Gabriel Hundeshagen, Javier Gonzalez, Samuel A. Knoedler, Martin Kauke-Navarro, Jasper Iske, Curtis L. Cetrulo, Maxime Jeljeli, Elena Hofmann, Max Heiland, Steffen Koerdt, Alexandre G. Lellouch

**Affiliations:** 1 Department of Oral and Maxillofacial Surgery, Charité—Universitätsmedizin Berlin, Corporate Member of Freie Universität Berlin, Humboldt-Universität zu Berlin, and Berlin Institute of Health, Berlin, Germany; 2 Medical Faculty, University of Heidelberg, Heidelberg, Germany; 3 Department of Hand, Plastic and Reconstructive Surgery, Burn Center, BG Trauma Hospital Ludwigshafen, Ludwigshafen, Germany; 4 Department of Plastic and Hand Surgery, University of Heidelberg, Ludwigshafen, Germany; 5 Cedars-Sinai Medical Center, Los Angeles, CA, United States; 6 College of Medicine, University of Arizona—Tucson, Tucson, AZ, United States; 7 Division of Plastic Surgery, Department of Surgery, Yale School of Medicine, New Haven, CT, United States; 8 Department of Cardiothoracic and Vascular Surgery, Charité—Universitätsmedizin Berlin, Corporate Member of Freie Universität Berlin, Humboldt-Universität zu Berlin, and Berlin Institute of Health, Berlin, Germany; 9 Vascularized Composite Allotransplantation Laboratory, Massachusetts General Hospital and Harvard Medical School, Boston, MA, United States; 10 Department of Plastic and Reconstructive Surgery, Shriners Children’s Boston, Boston, MA, United States

**Keywords:** vascularized composite allotransplantation, immunosuppression, tacrolimus, mycophenolate mofetil, antithymocyte globulin, infection prophylaxis, graft survival, systematic review

## Abstract

**Introduction:**

Vascularized composite allotransplantation (VCA) has achieved significant clinical success, but lifelong immunosuppression remains essential to prevent rejection. Despite potent regimens, including tacrolimus, mycophenolate mofetil, and steroids, rejection episodes frequently occur within the first postoperative year. The side effects of immunosuppressive drugs must be carefully balanced against the risks of insufficient therapy. This review specifically aims to evaluate current immunosuppressive regimens and infection prophylaxis in VCA to identify evidence based approaches that attempt to mitigate rejection, prevent infections, and improve long-term graft survival.

**Methods:**

A systematic review was conducted across PubMed/MEDLINE, EMBASE, and Web of Science databases, adhering to PRISMA 2020 guidelines. Inclusion criteria focused on studies reporting immunosuppressive regimens, dosages, and infection prophylaxis in VCA surgery. Non-VCA, animal, feasibility studies, and non-English publications were excluded.

**Results:**

Of 1,150 screened articles, 42 met inclusion criteria. Upper extremity and facial VCAs represented 50% and 29% of cases, respectively, with traumatic amputation as the primary indication (37%). Antithymocyte globulin was the most common induction drug, while tacrolimus, mycophenolate mofetil, and steroids were predominant for maintenance therapy in 33% and 11% of cases, respectively. Infection prophylaxis was used in 31% of cases. Drug dosages varied widely, and no standardized immunosuppressive protocols were identified.

**Conclusion:**

Current immunosuppressive strategies in VCA lack standardization, leading to variability in outcomes and increased risks. Infection prophylaxis remains underutilized despite recipient vulnerability. There is a critical need for standardized and tailored guidelines to optimize immunosuppressive therapy and infection control, ensuring graft survival and improved patient outcomes.

## 1 Introduction

VCA is an innovative branch of transplant surgery aimed at restoring form and function using composite allografts composed of skin, muscle, vasculature, and nerves (1). Moving beyond conventional reconstructive limitations, VCA offers improved aesthetic, functional, and psychological outcomes for patients with severe injuries or extensive tissue loss (2). Since the late 1990s, procedures such as face, abdominal wall, and hand transplants have shown promising long-term results (3).

However, the unique tissue composition of VCA increases the risk of strong immune responses, with around 85% of recipients experiencing acute rejection episodes (4). While skin tissue immunogenicity has been well-documented, recent findings highlight the oral and nasal mucosa as additional triggers for rejection (5, 6). Consequently, lifelong immunosuppression remains essential to prevent rejection and ensure transplant survival (1).

Research into VCA rejection mechanisms continues, with ongoing efforts to achieve immunotolerance (7). Currently, evidence-based care relies on combination immunosuppressive therapies, including corticosteroids (STR), tacrolimus (TAC), and mycophenolate mofetil (MMF), alongside anti-infectious prophylaxis (8). Despite advancements, limited data exist on optimal drug combinations, dosages, and long-term outcomes.

This review aims to consolidate current evidence on immunosuppressive regimens and anti-infectious prophylaxis in VCA surgery, offering insights to optimize patient care and improve surgical outcomes.

## 2 Methods

This systematic review followed the Preferred Reporting Items for Systematic Reviews and Meta-Analyses (PRISMA) 2020 guidelines. A narrative synthesis was selected due to the heterogeneity in outcome measures, rendering a meta-analysis unsuitable.

### 2.1 Systematic search

A comprehensive literature search was conducted across PubMed/MEDLINE, EMBASE, and Web of Science databases up to September 30th, 2024. The search strategy included two components combined using the Boolean operator “AND”. Search terms encompassed variations of “VCA” and “immunosuppression” and “immune tolerance”. The term “immune tolerance” was included as it is a key goal in VCA research and highly relevant to optimizing both immunosuppressive and infection prevention strategies. Moreover, “face transplantation” was included as a specific term due to its extensive and well-characterized immunological and infection-related literature, whilst “hand transplantation” was captured through broader VCA-related search terms that encompass upper-extremity transplants without requiring a separate keyword. Eligible articles had to be in English and accessible as full-text. Exclusion criteria included cadaver studies, animal studies, *in vitro* studies, and non-original data such as reviews or meta-analyses.

**Figure 1:**
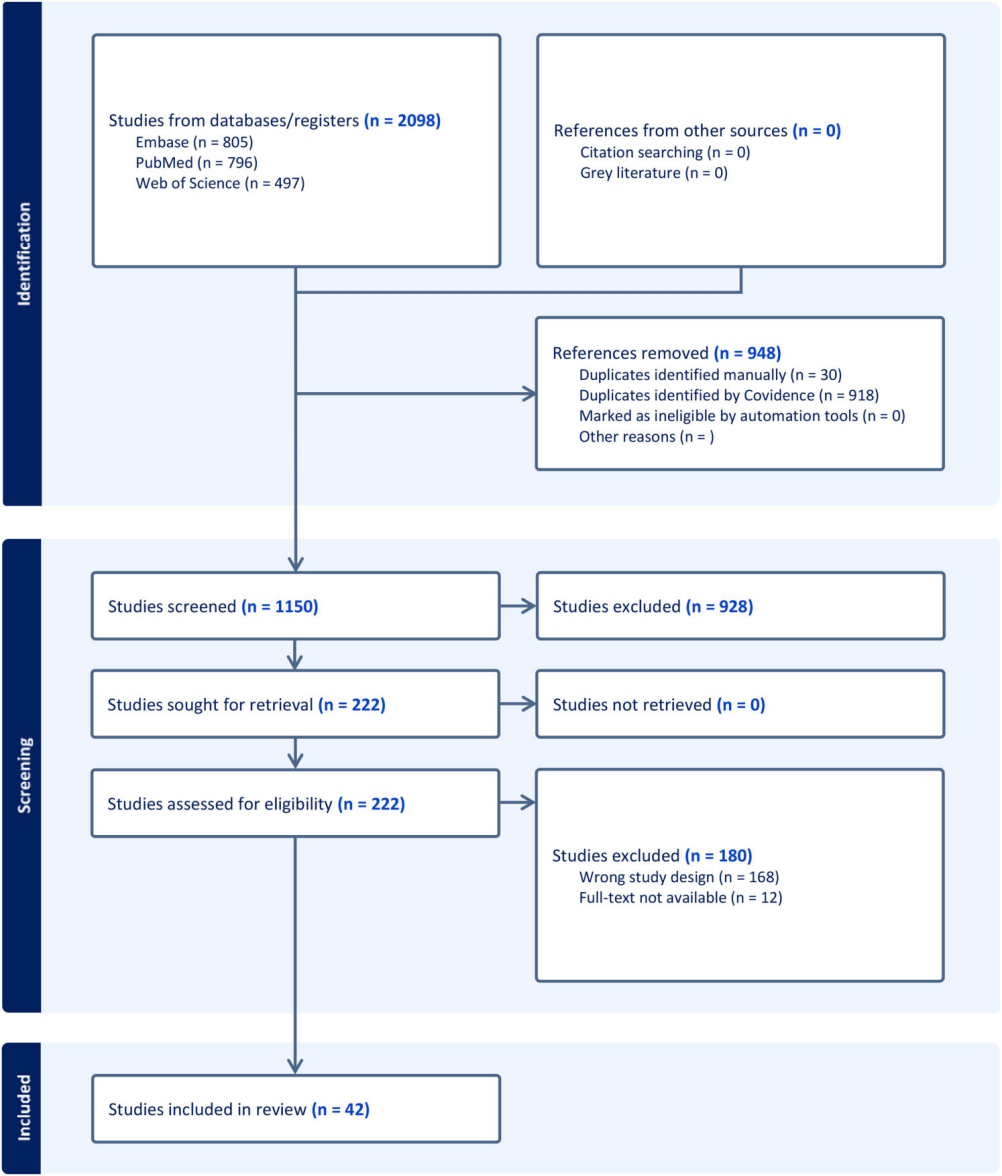
PRISMA 2020 flowchart.

Titles and abstracts were independently screened by two reviewers (T.S. and T.N.), followed by a full-text evaluation of eligible articles. If multiple studies analyzed the same patient cohort, the study with the most comprehensive immunosuppressive data and longest follow-up was selected. Discrepancies were resolved by consulting a third reviewer (L.K.). Detailed search strategies are provided in Supplementary Digital Content 1, and the PRISMA flowchart outlining the selection process is displayed in Figure 1.

### 2.2 Quality assessment

Study quality was evaluated using the Newcastle-Ottawa Scale and the Level of Evidence (LOE) system. The NOS assessed selection, comparability, and outcomes, assigning up to nine stars. Higher scores indicated better study quality and lower risk of bias. The LOE provided a hierarchical ranking based on methodological rigor, with systematic reviews and meta-analyses from randomized controlled trials ranked as Level I evidence.

### 2.3 Data extraction

Two reviewers independently extracted key variables, including DOI, author, study title, year, region, institution, study design, sample size, recipient and donor demographics, follow-up length, VCA type, indication, immunosuppressive protocols, infection prophylaxis, infectious complications, and outcomes. Data extraction details are available in Supplementary Table S1.

## 3 Results

### 3.1 General study characteristics

The systematic literature search identified 1,150 articles, with 42 (4%) meeting inclusion criteria. Given the limited number of global VCA cases and recurring reports on the same patients, studies were organized by individual cases, resulting in 102 unique cases. Publications spanned from 1999 to 2024, with case reports (66%) and case series (32%) being the predominant study types. One retrospective and one prospective cohort study (2% each) were included. Most studies (97%) were classified as LOE IV, with an average NOS score of 4.4 ± 0.5.

Among the recipients, 14% were female, and 86% were male, with ages ranging from 17 to 65 years (mean 46.6 ± 22.7 years). Donor ages ranged from 13 to 65 years (mean 38.4 ± 11.6 years). The follow-up period varied between 47 days and 19 years (mean 32.8 ± 35 months). The majority of studies were conducted in the United States (45%), followed by France (14%) and Austria (7%).

### 3.2 Indications for VCA

Face transplants (fVCA) represented 29% of cases, upper extremity (UE) transplants 50%, abdominal wall transplants 15%, tracheal transplants 5% and penile transplants 1%. The indication for fVCA was ballistic trauma in 46% of fVCA cases, burns in 39%, and animal attacks and neoplastic conditions in 7.1%, respectively. The indication for UE VCA was traumatic amputation in 42% of UE VCA cases, burns in 33%, and iatrogenic amputation due to sepsis or tissue ischemia in 3.9%, respectively. In 17% of UE VCA cases the indication was not reported. The indications for abdominal wall VCA were Gardner syndrome in 33%, trauma in 20%, intestinal motility disorders, including Hirschsprung disease and intestinal pseudo-obstruction, in 20%, gastroschisis in 13%, and Churg-Strauss vasculitis and significant abdominal wall scarring due to high-output small bowel enterocutaneous fistula in 6.7%, respectively. Further demographic and indication details are provided in Table 1.

**Table 1:** General study information.

Author	Year	Region	Study type	Sample size	Recipient age	Recipient sex	Donor age	Donor sex	VCA type	Indication	Follow-up
Dubernard	1999	France	CR	1	48	m	41	m	Unilateral forearm	Traumatic amputation (circular saw accident)	6 mo
Schneeberger	2006	Austria	CR	1	48	m	N/A	N/A	Bilateral hand	Traumatic amputation at wrist level	5 y
Bonatti	2007	Austria	CR	1	35	m	N/A	N/A	Bilateral forearm	Electrical burn	24 mo
Dubernard	2007	France	CR	1	38	f	46	f	Face	Dog bite	18 mo
Schneeberger	2008	USA	CS	3	P1: 22, P2: 32, P3: 36	m	N/A	N/A	Unilateral hand	Traumatic amputation	P1: 57 mo; P2: 65 mo; P3: 73 mo
Ravindra	2008	USA	CS	2	P1: 37, P2: 54	m	N/A	N/A	Unilateral hand	P1: firecracker accident, P2: traumatic amputation (industrial press accident)	N/A
Schneeberger	2009	USA	CS	3	P1: 54, P2: 46, P3: 31	P1: m, P2: f, P3: m	N/A	N/A	P1: unilateral hand, P2: bilateral hand, P3: bilateral hand	Traumatic amputation of proximal, mid or distal forearm	P1: 19 mo; P2: 18 mo; P3: 7 mo
Selvaggi	2009	USA	CS	14	10 adult, 4 pediatric	N/A	N/A	N/A	Abdominal wall and, multivisceral	Intestinal motility disorders, vasculitis, trauma	N/A
Delaere	2010	Belgium	CR	1	55	f	N/A	m	Trachea	Blunt trauma (car accident)	1 y
Siemionow	2010	USA	CR	1	45	f	N/A	N/A	Face	Ballistic trauma	8 mo
Lantieri	2011	France	Prospective cohort study	5	P1: 29, P2: 39, P3: 27, P4: 37, P5: 33	m	P1: 65, P2: N/A, P3: 43, P4: 59, P5: 55	m	Face	P1: NF1, P2,4: burn, P3,5: ballistic trauma	38 mo
Cavadas	2011	Spain	CR	1	29	m	25	m	Bilateral forearm	Electrical burn	2 y
Barett	2011	Spain	CR	1	30	m	41	m	Face	Ballistic trauma	120 d
Pomahac	2011	USA	CR	1	59	m	60	m	Face	Electrical burn	1 y
Delaere	2012	Belgium	CS	4	P1: 26, P2: 45, P3: 17, P4: 64	P1: m, P2: f, P3: m, P4: m	N/A	N/A	Trachea	N/A	6 w to 4 mo
Pei	2012	China	Retrospective cohort study	12	P1: 39, P2: 27, P3: 25, P4: 24, P5: 37, P6: 19, P7: 50, P8: 43, P9: 52, P10: 37, P11: 19, P12: 38	m	P1: 29, P2: 25, P3: 30, P4: 29, P5: 35, P6: 20, P7: 48, P8: 35, P9: 50, P10: 42, P11: 24, P12: 23	P1: m, P2: m, P3: m, P4: m, P5: m, P6: m, P7: m, P8: m, P9: m, P10: m, P11: m, P12: m	P1: right wrist, P2: right wrist, P3: right wrist, P4: right thumb, P5: double proximal forearm, P6: double wrist, P7: left proximal forearm, P8: right distral forearm, P9: double proximal forearm, P10: right proximal forearm, P11: left palm, P12: right wrist	P1: traumatic amputation, P2: explosion, P3: traumatic amputation, P4: explosion, P5: explosion;P6: thermal injury (low temperature), P7: explosion, P8: explosion, P9: machine injury, P10: machine injury, P11: machine injury, P12: traumatic amputation	P1: 10 y; P2: 2 y; P3: 1 y; P4: 1 y; P5: 9 y; P6: 8 y; P7: 7 y; P8: 2 y; P9: 6 y; P10: 1 y; P11: 2 y; P12: 2 y
Kamińska	2014	Poland	CS	5	P1: 56, P2: 28, P3: 34, P4: 29, P5: 30	P1: f, P2: m, P3: m, P4: m, P5: m	P1: 47, P2: 50, P3: 41, P4: 53, P5: 51	P1: f, P2: m, P3: m, P4: f, P5: f	Hand	N/A	74 mo
Chandraker	2014	USA	CR	1	45	f	45	f	Face	Chemical burn (lye)	N/A
Petruzzo	2015	France	CR	1	27	m	N/A	N/A	Face	Pyrotechnic accident	N/A
Petruzzo	2015	France	CS	5	P1: 33, P2: 21, P3: 27, P4: 29, P5: 21	P1: m, P2: m, P3: f, P4: m, P5: m	P1: 18, P2: 45, P3: 40, P4: 29, P5: 18	N/A	Bilateral hand	P1: explosion, P2: crush injury, P3: electrical burn, P4: burn injury, P5: explosion	N/A
Kanitakis	2015	France	CS	2	P1: 38, P2: 27	f	P1: 46, P2: 40	f	P1: face, P2: bilateral hand	P1: dog attack, P2: electrical burn	P1: 6 y, P2: 8.4 y
Kuo	2016	Taiwan	CR	1	45	m	37	m	Unilateral hand	Traumatic amputation	9 mo
Kim	2015	USA	CR	1	27	f	N/A	N/A	Unilateral hand	N/A	750 d
Diaz-Siso	2015	USA	CS	1	65	m	N/A	N/A	Bilateral upper extremity	Iatrogenic amputation due to septic shock	28 to 58 mo, median: 34 mo
Selber	2016	USA	CR	1	55	m	33	m	Face	Iatrogenic calvaria osteoradionecrosis post removal of scalp tumor	1 y
Iyer	2017	India	CS	2	N/A	N/A	N/A	N/A	Bilateral hand	N/A	1 y
Özkan	2017	Turkey	CS	5	P1: 19, P2: 35, P3: 26, P4: 54, P5: 22	m	P1: 37, P2: 19, P3: 42, P4: 31, P5: 34	m	Face	P1–2: burn injury, P3–5: ballistic trauma	11 mo to 2 y
Kwon	2018	Korea	CR	1	35	m	49	m	Unilateral forearm	Trauma	47 d
Gelb	2018	USA	CR	1	41	m	26	m	Face	Burn injury	24 mo
Fallahian	2018	USA	CR	1	42	m	Suitable match	Suitable match	Bilateral upper extremity	N/A	3 y
Cetrulo	2018	USA	CR	1	64	m	27	m	Penis	Subtotal penectomy due to penile cancer	6 mo
Cendales	2018	USA	CR	1	54	m	N/A	m	Unilateral forearm	Traumatic amputation (meat grinder accident)	20 mo
Krezdorn	2019	USA	CS	6	P1: 25, P2: 59, P3: 30, P4: 44, P5: 39, P6: 33	P1: m, P2: m, P3: m, P4: m, P5: f, P6: m	P1: 48, P2: 60, P3: 31, P4: 56, P5: 23, P6: 51	Suitable match	Face	P1–3: electrical burn, P4: chemical burn (acid), P5–6: ballistic trauma	45 to 112 mo, mean: 76,4 ± 23.5 SD
Hautz	2020	Austria	CS	5	P1: 47, P2: 41, P3: 23, P4: 55, P5: 55	m	N/A	N/A	P1: bilateral distal forearm, P2: bilateral proximal forearm, P3: bilateral mid forearm, P4: unilateral wrist, P5: bilateral wrist	P1 + 3 explosion, P2: electrical burn, P4: timber machine accident, P5: car accident	P1: 19 y, P2: 16 y, P3: 13 y, P4: 7 y, P5: 5 y
Govshievich	2021	Canada	CR	1	64	m	Younger than recipient	m	Face	Ballistic trauma	18 mo
Roy	2020	Canada	CR	1	65	m	N/A	N/A	Face	Ballistic trauma	N/A
Atia	2020	USA	CR	1	37	m	13	m	Abdominal wall	Small-bowel fistulas	1 y
Azoury	2021	USA	CR	1	40	f	N/A	N/A	Bilateral hand	Tissue ischemia	1 y
Lee	2023	South Korea	CR	1	62	m	N/A	N/A	Unilateral hand	Traumatic amputation	1 y
Ozmen	2023	Turkey	CR	1	20	f	29	f	Face	Ballistic trauma	56 mo
Murakami	2023	USA	CS	2	P1: 57, P2: 60	f	N/A	N/A	Face	P1: Animal attack, P2: N/A	P1: 48 w, P2: 1 y and 9 mo
Zaccardelli	2024	USA	CR	1	65	m	N/A	N/A	Bilateral upper extremety	Iatrogenic amputation due to urosepsis with ARDS	12 y

VCA, vascular composite allograft; CR, case report; CS, case series; P, patient; m, male; f, female; d, day; w, week; mo, month; y, year; NF, neurofibromatosis; N/A, not available.

### 3.3 Induction immunotherapy

All VCA cases (*n* = 102, 100%) received induction immunotherapy (9–53). The most common drugs were antithymocyte globulin (ATG, *n* = 52, 51%), tacrolimus (TAC, *n* = 44, 43%), mycophenolate mofetil (MMF, *n* = 42, 41%), and steroids (STR, *n* = 51, 50%), with prednisone (PDN, *n* = 50, 49%) being the preferred option. Quintuple induction was used in *n* = 5 (5%) cases, combining ATG, TAC, MMF, PDN, and one additional agent (e.g., daclizumab, rituximab, everolimus, plasmapheresis, or bone marrow infusion; *n* = 1, 1% each) (9, 20, 26, 31, 32). Quadruple therapy was chosen in *n* = 24 (24%) cases, most commonly ATG, TAC, MMF, and PDN (*n* = 18, 18%). Other combinations included basiliximab (*n* = 3, 3%), IL2 (*n* = 2, 2%), or belatacept (BLC, *n* = 1, 1%) (11, 12, 24, 27, 29, 39, 40, 44). Triple therapy was used in *n* = 21 (21%) cases, with ATG, TAC, and PDN (*n* = 10, 10%) as the most common combination (12, 19, 23, 33, 34, 36, 38, 42). Double therapy (*n* = 5, 5%) included combinations such as ATG and PDN (*n* = 2, 2%) or TAC paired with azathioprine (AZP), MMF, or alemtuzumab (ALZ) (*n* = 1, 1% each) (15, 17, 22, 48). Single-agent induction (*n* = 41, 40%) featured ALZ (*n* = 18, 18%), ATG (*n* = 12, 12%), basiliximab (BSX, *n* = 9, 9%), and cyclosporin A (CSA, *n* = 2, 2%) (10, 12, 14, 18, 25, 28, 30, 35, 40, 41, 43, 46, 47, 49, 50).

Focusing on drug dosages, ATG dosage varied from 75 to 100 mg/day (*n* = 10, 10%) to 1–3 mg/kg/day (*n* = 35, 34%). Higher doses (6 mg/kg, 7.14 mg/kg, and 10 mg/kg) were used in *n* = 1 (1%) case each (26, 44, 48). TAC levels ranged from 6 to 8 ng/mL (*n* = 1, 1%) to 10–15 ng/mL (*n* = 4, 4%). Dosages included 0.2 mg/kg/day (*n* = 2, 2%) and 5 mg/day (*n* = 10, 10%) (9, 11, 21, 24, 27, 34, 36, 40). MMF was administered at 2,000 mg/day (*n* = 13, 13%), 1,500 mg/day (*n* = 1, 1%), 1,000 mg/day (*n* = 6, 6%), 750 mg/day (*n* = 2, 2%), and 500 mg/day (*n* = 1, 1%) (9, 11, 12, 20, 23, 24, 29, 37, 39, 40, 42, 44, 45). STR dosages included 1,500 mg/day (*n* = 1, 1%), 1,000 mg/day (*n* = 11, 11%), 500 mg/day (*n* = 11, 11%), 250 mg/day (*n* = 6, 6%), and 100 mg/day, 50 mg/day, and 5 mg/day (*n* = 1, 1% each). ALZ was administered at 30 mg (*n* = 2, 2%), BSX at 20 mg (*n* = 3, 3%), CSA at 400 mg (*n* = 2, 2%), and rituximab (RXM) at 1,000 mg (*n* = 1, 1%) (12, 17, 22, 26, 35, 36, 43). Further details are provided in Table 2.

**Table 2:** Summary of immunosuppressive schemas, including induction and maintenance immunosuppression.

Author	Year of publication	VCA type	Length of follow-up	Induction immunosuppression	Maintenance immunosuppression	Rejection
Dubernard	1999	Unilateral forearm	6 mo	ATG (75 mg/day for 10 days), TAC (blood level of 10 ng/mL to 15 ng/mL during POM 1), MMF (2 g/day), PDN (250 mg on POD 1, tapered to 20 mg/day), Daclizumab (CD25 monoclonal antibody) on POD 26 and POD 100	TAC (maintenance blood level of 5 ng/mL to 10 ng/mL), MMF (2 g/day), PDN (POM 3: 20 mg/day, POM 6: 15 mg/day)	AR: POW 8–9
Schneeberger	2006	Bilateral hand	5 y	ATG	TAC (maintenance blood level of 10 ng/mL), MMF (2 g/d), PDN (5 mg), POM 30: SRL (4–8 ng/mL), TAC (3–4 ng/mL), POM 36: STR weaned, POM 39: TAC discontinued, leaving patient on SRL and MMF	AR: POD 55, POD 188, POM 48
Bonatti	2007	Bilateral forearm	24 mo	N/A	N/A	3 AR episodes
Dubernard	2007	Face	18 mo	ATG for 10 days, TAC (blood concentrations between 10 and 15 ng/mL during first month), MMF (2 g/d), PDN (250 mg on day 1, 100 mg on day 2 and 60 mg/d until POD 12 with gradual taper)	TAC (p.o.), MMF (2 g/d), PDN (10 mg/d p.o.), topical TAC and STR; POM 11: SRL (maintenance blood level of 8 to 12 ng/mL), infusion of fresh frozen plasma for 4 days, high dose of i.v. immune globulin	AR: POD 18, POD 214; POD 18: candida stomatitis due to Candida albicans
Schneeberger	2008	Unilateral hand	P1: 57 mo; P2: 65 mo; P3: 73 mo	P1: ATG, P2: Basiliximab, P3: Basiliximab	P1: TAC, MMF, STR, P2: TAC, MMF, STR, P3: SRL, MMF, steroids	P1: AR: POM 43 months PO; P2: AR: POM 3, POM 27; P3: POD 10, POD 21, POD 50, POD 77
Ravindra	2008	Unilateral hand	N/A	P1: 20 mg of basiliximab pre-op and on POD 4, P2: 30 mg alemtuzumab intra-op	P1: TAC (maintenance blood level of 15–20 ng/mL until POM 6), MMF (1 g BID), PDN (tapered to 7.5 mg/d by POM 6; POY 8: TAC (maintenance blood level of 6–8 ng/mL), MMF (1 g BID), PDN (7.5 mg/d), P3: TAC (maintenance blood level of 10–15 ng/mL until POM 6, then 8–10 ng/mL), MMF, PDN (3 doses peri-op until POD 3)	P1: AR: POM 2, POM 5, POM 7; P2: AR
Schneeberger	2009	P1: unilateral hand, P2: bilateral hand, P3: bilateral hand	P1: 19 mo; P2: 18 mo; P3: 7 mo	TAC (5 mg p.o.), MMF (2 g p.o.), basiliximab (1 amp. i.v.) and PDN (1 g i.v.). POD 1: TAC (5 mg p.o. BID), MMF (2 g/d), MPDN (0.5 g intravenously); POD 2: TAC (5 mg p.o. BID), MMF (2 g/day), POD 3 to 7 TAC adjusted to 20 ng/mL	P1: TAC (maintenance blood level of 10–15 ng/mL until POM 6, then 5–10 ng/mL) MMF (p.o.); POY 1: TAC (2 mg BID), MMF (500 mg BID); P2: TAC (switched to sirolimus on POD 190), MMF, STR; P3: TAC, MMF, STR	P1: AR: XXXX POM 18; P2: AR: POD 120, POD 221; P3: AR: POD 30, POD 170
Selvaggi	2009	Abdominal wall or multivisceral	N/A	Alemtuzumab	STR-free TAC based maintenance	POD 1 and 6: graft losses to vascular thrombosis; AR: 4 episodes resolved
Delaere	2010	Trachea	1 y	TAC (12 to 15 ng/mL), azathioprine (100 mg/day), corticosteroids (0.4 mg/kg)	TAC (6 mg/d p.o.), azathioprine (100 mg/d), PDN (4 mg/d)	Rejection of skin graft post discontinuation of immunosuppressive therapy
Siemionow	2010	Face	8 mo	ATG	TAC, MMF, STR	AR
Lantieri	2011	Face	38 mo	ATG (1 mg/kg/day for 10 days), TAC (blood concentration of 10–13 ng/mL in POM 3), MMF (2 g/day), PDN (500 mg on day 1, 250 mg on day 2, 120 mg on day 3, 60 mg/day for 7 days, tapered to 10 mg/day)	TAC (maintenance blood level of 8–10 ng/mL), MMF, PDN; P3, 4, 5: extracorporeal photopheresis (BIW for 1 m, MIW for 3 m)	P1: AR: POD 28, POD 64; P2: N/A; P3: AR: POD 0; P4: No rejection episode; P5: AR: POD 5
Cavadas	2011	Bilateral forearm	2 y	Alemtuzumab (30 mg i.v.), PDN (POD 1: 500 mg, POD 2: 250 mg, then discontinued)	TAC (switched to sirolimus on POD 332), MMF (2 g/d)	AR: POM 6, POM 13, POM 26
Barett	2011	Face	120 d	ATG (i.v. 2 mg/kg) 2 h pre-op, PDN (1 g)	TAC (maintenance blood level of 10–15 ng/mL), MMF (2 g/d p.o.), PDN (1 mg/kg/d i.v. tapered to 10 mg/d)	AR: POD 3, POD 7, POD 28, POD 75, POM 3
Pomahac	2011	Face	1 y	ATG (1.5 mg/kg), MMF (1000 mg) pre-op, PDN (500 mg) intra-op	TAC (maintenance blood level of 10–15 ng/mL), MMF (2 g/d), PDN (tapered to 20 mg by POD 5); POD 106: TAC (5 mg BID), MMF (720 mg), PDN (10 mg); POM 9: TAC (1 mg BID, maintenance blood level of 5–8 ng/mL), POD 360: PDN discontinued	AR: POD 17, POD 74, POD 107
Delaere	2012	Trachea	6 w to 4 mo	Triple immunosuppression (i.v.)	TAC (6 mg), azathioprine (100 mg), PDN (4 mg)	P1: AR: POM 2, POM 4, POM 6.5, POM 7, P2: CR
Pei	2012	P1: right wrist, P2: right wrist, P3: right wrist, P4: right thumb, P5: double proximal forearm, P6: double wrist, P7: left proximal forearm, P8: right distral forearm, P9: double proximal forearm, P10: right proximal forearm, P11: left palm, P12: right wrist	P1: 10 y; P2: 2 y; P3: 1 y; P4: 1 y; P5: 9 y; P6: 8 y; P7: 7 y; P8: 2 y; P9: 6 y; P10: 1 y; P11: 2 y; P12: 2 y	P1: ATG (100 mg/d), TAC (5 mg/d), MMF (750 mg/d) PDN (1 g/d), P2: ATG (100 mg/d) TAC (5 mg/d), MMF (750 mg/d), PDN (1 g/d), P3: CTX (400 mg/d), P4: CTX (400 mg/d), P5: ATG (100 mg/d), TAC (5 mg/d), MMF (500 mg/d), PDN (1 g/d), P6: ATG (80 mg/d), TAC (5 ng/mL), PDN (800 mg/d), P7: ATG (80 mg/d), TAC (5 ng/mL), PDN (800 mg/d), P8: ATG (80 mg/d), TAC (5 ng/mL), PDN (800 mg/d), P9: ATG (80 mg/d), TAC (5 ng/mL), PDN (800 mg/d), P10: N/A, P11: N/A, P12: N/A	P1: TAC (3 mg/d), MMF (stopped after 6 m), PDN (5 mg/d); P2: N/A; P3: TAC (1 mg/d), MMF (1 g/d), PDN (10 mg/d); P4: TAC (1 mg/d), MMF (1 g/d), PDN (10 mg/d), P5: TAC, MMF, PDN, P6: TAC (3 mg/d), MMF (stopped after 6 m), PDN (5 mg/d); P7: TAC (3 mg/d), MMF (stopped after 6 m), PDN (5 mg/d); P8: TAC (3 mg/d), MMF (stopped after 6 m), PDN (5 mg/d); P9: TAC (3 mg/d), MMF (stopped after 6 m), PDN (5 mg/d); P10: N/A; P11: N/A; P12: N/A	P1: AR every POY; P2: AR: POM 15; P3: AR once post-op; P4: 1 AR; P5: AR every POY; P6: AR every POY; P7: AR every POY; P8: AR: POM 6, POY 2; P9: AR: POY 1, POY 3, POY 5, POY 6; P10: POM 7; P11: POW 4, POW 8, POY 2; P12: POY 2
Kamińska	2014	Hand	74 mo	Basiliximab	TAC (maintenance blood levels of 10–15 ng/mL), MMF (2 g/d), PDN (20–40 mg/d)	AR in P1: 1; P2: 2; P3: 2; P4: 7; P5: 2
Chandraker	2014	Face	N/A	ATG 1.5 mg/kg/day for 4 days, MMF 2 g/d, STR, TAC (2 mg; goal of 10 mg). POD 1: Plasmapheresis, 10 g immunoglobulin (150 mg/kg)	TAC, MMF	AR: POD 12, POD 15, POD 19
Petruzzo	2015	Face	N/A	ATG	TAC (maintenance blood levels of 5–10 ng/mL), MMF (2 g/d); POD 4: PDN (5 mg/d), infusion of donor bone-marrow cells; At present: everolimus (3 mg/d), STR (16 mg/d), extracorporal photochemotherapy	AR: POD 41, POD 103, POD 186, POD 239, POD 474, POD 527, POD 540, POD 931
Petruzzo	2015	Bilateral hand	N/A	P1: ATG 1.25 mg/kg/d for 10 days, P2–5: ATG (3 mg/kg on POD 1 and 2 mg/kg on POD 2); PDN 250 mg on POD 1 (tapered: 20 mg/d), TAC (0.1 mg/kg since POD 2 (blood level of 10 and 15 ng/mL) and MMF (2 g/d)	P1, 2, 4, 5: TAC (maintenance blood level of 5–10 ng/mL tapered to 5–8 ng/mL), PDN (5 mg/d), MMF (2 g/d), P3: TAC (maintenance blood level of 4–7 ng/mL), PDN (5 mg/d), sirolimus (maintenance blood level of 6–10 ng/mL), MMF (1 g/d); POM 26: P5 switched from TAC to sirolimus for 14 m	P1: AR: POD 53, POD 72; P2: POD 57, POD 86, POD 2,759; P3: POD 16, POD 271, POD 635, POD 951, POD 1,365, POD 1,855; P4: POD 65; P5: POD 10, POD 350, POD 560
Kanitakis	2015	P1: face, P2: bilateral hand	P1: 6 y, P2: 8.4 y	P1: TAC, MMF, PDN, ATG, POD 4 and 11: bone marrow infusion, P2: N/A	P1: sirolimus (from POM 11 onwards), MMF, PDN, extracorporeal photochemotherapy over 2 years; P2: TAC, MMF, STR	P1: no rejection episodes; P2: several AR episodes
Kuo	2015	Unilateral hand	9 mo	ATG (1.25 mg/kg for 10 days), PDN (pre-op: 500 mg, POD 0: 250 mg, POD 1: 125 mg, tapered to 10 mg); POD 1: TAC (blood concentrations of 10–15 ng/mL until POM 6, 8 to 10 ng/mL after 6 months, and then maintaining serum levels of 5–8 ng/mL).	TAC, MMF (2 g/d), PDN (10 mg/d)	AR: POM 3.5, POD 105
Kim	2015	Unilateral hand	750 d	TAC, MMF, ATG, Everolimus, PDN	TAC, MMF, ATG, Everolimus, PDN	AR: POD 717
Diaz-Siso	2015	Bilateral upper extremity	28 to 58 mo, median: 34 mo	ATG (1.5 mg/kg for 4 days), PDN (500 mg/day with gradual taper, MMF (1000 mg pre-op)	TAC (maintenance blood levels of 10–15 ng/mL for 3–21 d), MMF (1,000 mg BID), PDN (20 mg on POD 5, tapered)	AR: POM 16, POM 26
Selber	2016	Face	1 y	ATG for (7.14 mg/kg cumulative dose), PDN (1,500 mg i.v.)	TAC (maintenance blood level of 7–10 ng/mL), MMF (1 g BID), PDN (5 mg/d p.o.) daily, topical TAC	AR: POW 11
Iyer	2017	Bilateral hand	1 y	ATG (1.5 mg/kg i.v.), TAC (0.05 mg/kg), PDN (500 mg i.v.), MMF (1,000 mg); POD 0: ATG (1.5 mg/kg), TAC (0.1 mg/kg BID), PDN (250 mg i.v.), MMF (1,000 mg BID); POD 1–5: ATG (1.5 mg/kg for 3 days), TAC (0.1 mg/kg BID), PDN (0.5 mg/kg/day p.o.), MMF (1,000 mg BID)	TAC, MMF (1,000 mg BID), PDN (0.5 mg/kg/d)	P1: POW2, POW4, POM 8, POM 9; P2: POM 1
Özkan	2017	Face	11 mo to 2 y	ATG (2.5 mg/kg/day intra-op), PDN (1,000 mg, tapered to 20 mg in POW 1); POD 4: TAC (0.2 mg/kg/day, blood concentration of 15 to 20 ng/mL); ATG stopped on POD 7–10	P1, 2, 3: TAC (maintenance blood levels of 15–20 ng/mL until POM 3, 7–10 ng/mL until POM 6), MMF (2 g/d), PDN (20 mg/d, tapered to 10 mg/d by POM 6); P4: modified protocol	AR: all patients between POM 6 and POY 5; P1: 12 AR in POM 12; P2: POY 1; P3: POM 15; P4: POM 24
Kwon	2018	Unilateral forearm	47 d	Basiliximab (IL-2 receptor blocker, 20 mg)	TAC (5 mg/d), MMF (750 mg/d), PDN (125 mg/d)	AR: POD 6, POD 47
Gelb	2018	Face	24 mo	ATG (6 mg/kg, total dose of 575 mg), PDN (tapered), rituximab (1 g), TAC (5 mg intra-op), MMF (1,000 mg)	TAC, MMF, PDN	No episodes of rejection
Fallahian	2018	Bilateral upper extremity	3 y	ATG (1.5 mg/kg)	TAC (maintenance blood level of 8–10 ng/mL), MMF (720 mg BID), PDN (10 mg/d), plasmapheresis prior to discharge	1 AR episode
Cetrulo	2018	Penis	6 mo	ATG, MMF, PDN	MMF, TAC, PDN (tapered)	AR: POD28, POD 32
Cendales	2018	Unilateral forearm	20 mo	ATG (1,5 mg/kg, 3 doses)	Belatacept (10 mg/kg, 2 doses), TAC (5 mg/kg, maintenance blood level of 10–15 ng/mL); POM 6: TAC switched to SRL (maintenance blood level of 8–12 ng/mL), MMF (1 g BID), PDN (tapered to 10 mg/d); POM 20: belatacept 5 mg/kg monthly, MMF (500 mg BID), PDN (10 mg/d)	AR: POM 8
Krezdorn	2019	Face	45 to 112 mo, mean: 76,4 ± 23.5 SD	P1,2,3,4: TAC, MMF, PDN, P5: TAC, MMF, P6: TAC, MMF, PDN, Belatacept	N/A	Number of AR: P1: 5, P2: 4, P3: 5, P4: 6, P5: 2, P6: 5; CR in P1: POM 25, P2: POM 68
Hautz	2020	P1: bilateral distal forearm, P2: bilateral proximal forearm, P3: bilateral mid forearm, P4: unilateral wrist, P5: bilateral wrist	P1: 19 y, P2: 16 y, P3: 13 y, P4: 7 y, P5: 5 y	P1, 2: ATG, P3, 4, 5: Alemtuzumab	P1, 2, 3, 5: TAC, MMF, STR; P4: TAC, MMF; belatacept in P5 (discontinued after 3 y in P2 and P3)	AR: all patients; in total 43 Ars; CR in POY 7 in P4
Govshievich	2020	Face	18 mo	ATG, TAC (blood concentration of 10 to 15 ng/mL), MMF, PDN	TAC (maintenance blood level of 10–15 ng/mL), MMF (same dose as in induction), PDN (tapered over 5 w)	AR episode
Roy	2020	Face	N/A	ATG (10 mg/kg i.v.), TAC, MMF (2 g/d), PDN (50 mg/d i.v., tapered to 25 mg)	TAC (maintenance blood level of 10–15 ng/mL until POM 6, then 8 ng/mL), MMF (1 g i.v. BID), PDN (50 mg/d i.v., tapered to 25 mg/d), POW 10: topical TAC added; basiliximab on POD 105, 133, 161 and 217	AR: POD 50, POD 56, POD 286
Atia	2020	Abdominal wall	1 y	ATG (1.5 mg/kg, 4 doses)	TAC (maintenance blood level of 15–18 ng/mL for 3 m), MMF (1,000 mg BID), PDN (20 mg/d, tapered)	AR episode
Azoury	2021	Bilateral hand	1 y	ATG (75 mg, 5 doses)	TAC, MMF (1 g/d), PDN, SRL	AR episode
Lee	2023	Unilateral hand	1 y	TAC (3 mg pre-op then 4 mg/d, blood concentration of 6–8 ng/mL), STR, basiliximab (20 mg BID pre-op and POD 4), PDN (100 mg intra-op)	TAC (maintenance level of 6–8 ng/mL), MMF (started on POD 14 at 1,000 mg/d), PDN (tapered to 10 mg/d by POD 17)	AR: POD 33, POD 41
Ozmen	2023	Face	56 mo	PDN (1,000 mg), ATG (100 mg), TAC (5 mg BID), MMF (1,000 mg BID)	TAC (2 doses at 8 mg), azathioprine (2 doses at 50 mg), PDN (40 mg/morning, 20 mg/evening)	AR: POD 26
Murakami	2023	Face	48 w	P1: ATG (POM 6: blood concentration of 6–8 ng/mL), MMF (1.500 mg/d), PDN (5 mg/d), started on “IL-2 protocol"	P1: TAC, MMF, PDN (discontinued at POM 4.5; P2: POM 6: TAC (maintenance blood level of 6–8 ng/mL), MMF (1,500 mg/d), PDN (5 mg/d), started on the “IL-2 protocol”. P2: 30% of the IL-2 dose from P1	P1: AR: POM 2, POM 17, POM 30, POM 47, POM 58
Zaccardelli	2024	Bilateral upper extremety	12 y	ATG (4 doses i.v.)	TAC (maintenance blood level of 10–15 ng/mL), MMF (1,000 mg BID), PDN (7.5 mg/D); TAC and MMF weaned to 5 ng/mL and 360 mg/d	AR: POM 26, POM 37

VCA, vascular composite allograft; CR, case report; CS, case series; P, patient; m, male; f, female; d, day; w, week; mo, month; y, year; NF, neurofibromatosis; ATG, antithymocyte globulin; TAC, tacrolimus; MMF, mycophenolic acid; PDN, prednisone; STR, steroids; AR, acute rejection; CR, chronic rejection; POD, postoperative day; POW, postoperative week; POM, postoperative month; POY, postoperative year; BID, bidaily; N/A, not available.

### 3.4 Maintenance immunotherapy

For maintenance therapy, TAC (*n* = 86, 84%), MMF (*n* = 69, 68%), and STR (*n* = 71, 70%) were commonly used, with PDN (*n* = 61, 60%) being the primary steroid (9–53). A sextuple regimen (*n* = 1, 1%) included TAC, MMF, PDN, everolimus (ERL), bone marrow infusion, and extracorporeal photochemotherapy (41). Quintuple therapy (*n* = 3, 3%) included combinations with SRL, BLC, ERL, and fresh frozen plasma (18, 24, 32). Quadruple therapy (*n* = 11, 11%) featured TAC, MMF, PDN, and IL2 (*n* = 1, 1%), BSX (*n* = 1, 1%), SRL (*n* = 2, 2%), or RXM (*n* = 2, 2%). Four cases (4%) received TAC, MMF, PDN, and extracorporeal photochemotherapy (9–15, 18, 19, 23–29, 34–38, 40–48, 50). Triple therapy (*n* = 54, 53%) commonly included TAC, MMF, and PDN (*n* = 41, 40%). Variations included TAC, PDN, and AZP (*n* = 6, 6%) or TAC, MMF, and BLC (*n* = 1, 1%) (18, 24, 32, 37, 38, 40, 48). Double therapy (*n* = 2, 2%) consisted of TAC and MMF (20, 45). No single-drug maintenance therapies were reported. Topical immunosuppression (*n* = 3, 3%) included TAC (*n* = 3, 3%) and PDN (*n* = 1, 1%) (24, 44, 48).

TAC target blood levels ranged from 4 to 7 ng/mL (*n* = 1, 1%) to 15–20 ng/mL (*n* = 4, 4%). Daily dosages included 1–10 mg/day, with 6 mg/day and 3 mg/day being the most common (*n* = 6, 6% each). SRL replaced TAC in *n* = 6 (6%) cases, with target levels between 6 and 12 ng/mL. ERL was used at 3 mg/day (*n* = 1, 1%) (41). MMF was typically administered at 2,000 mg/day (*n* = 24, 24%), followed by 1,000 mg/day (*n* = 10, 10%). Lower doses of 1,500 mg/day, 750 mg/day, and 500 mg/day were seen in isolated cases (9, 13–15, 17, 23, 24, 29, 30, 34, 37, 38, 42, 43, 48). BLC was administered at 20 mg/kg/month (*n* = 1, 1%), and AZP at 100 mg/day (*n* = 6, 6%) (18, 21, 22, 39). All cases receiving STR in induction therapy had tapered regimens. Further taper details are outlined in Table 2 (12–53).

### 3.5 Immunosuppressive combinations and rejection rates in various VCA types

Acute rejection (AR) occurred in 93% (*n* = 95) of VCA cases, with allograft loss in 7% (*n* = 7). A total of 255 AR episodes were reported [median (IQR): 2.5 (1–3.5) ARs per case].

In upper extremity VCAs, the most common induction regimen was ATG, TAC, MMF, and PDN (*n* = 9, 9%), resulting in 38 AR episodes [median (IQR): 4.2 (2.9–4.6) ARs per case]. Single-drug induction with ATG (*n* = 5, 5%) led to 8 AR episodes [median (IQR): 1.6 (1.0–2.0) ARs per case]. For maintenance, TAC, MMF, and STR (*n* = 34, 33%) resulted in 125 AR episodes [median (IQR): 3.6 (1–2.1) ARs per case]. Enhanced regimens, including SRL (*n* = 4, 4%), SRL + BLC (*n* = 1, 1%), ATG + ERL (*n* = 1, 1%), and plasmapheresis (*n* = 1, 1%), reported 15 AR episodes [median (IQR): 2.1 (1–2.5) ARs per case]. Across the main indications for UE VCA, in traumatic amputation the most common induction regimens were ATG monotherapy (*n* = 5, 5%), BSX monotherapy (*n* = 8, 8%) and ATG, TAC, MMF, PDN (*n* = 4, 4%), whereas in burns the most common induction regimens were ATG, TAC, MMF, PDN (*n* = 4, 4%) and ALZ monotherapy (*n* = 4, 4%). For maintenance, TAC, MMF, STR were the most common regimen in UE VCA cases due to traumatic amputation (*n* = 10, 10%) and in burns (*n* = 7, 7%).

In fVCAs, ATG, TAC, MMF, and STR (*n* = 9, 9%) were the most common induction drugs, resulting in 11 AR episodes [median (IQR): 1.5 (1–2) ARs per case]. Enhancements with RXM, bone marrow infusion, or plasmapheresis (*n* = 1 each, 1%) resulted in 3 AR episodes [median (IQR): 1.0 (0.8–2.3) ARs per case]. Maintenance therapy with TAC, MMF, and STR (*n* = 11, 11%) led to 25 AR episodes [median (IQR): 2.5 (1.0–3.0) ARs per case]. Enhanced combinations, including BSX (*n* = 1, 1%), IL2 (*n* = 2, 2%), ERL, fresh frozen plasma, or photopheresis (*n* = 1 each, 1%), resulted in 20 AR episodes [median (IQR): 2.5 (2.1–2.9) ARs per case]. In the main indications for fVCA, induction with ATG, TAC, MMF, STR (*n* = 5, 5%) was the most common in ballistic trauma and burns (*n* = 4, 4%), followed by TAC, MMF, STR (*n* = 2, 2%) in ballistic trauma and burns (*n* = 4, 4%). For maintenance the most common regimen was TAC, MMF, STR (*n* = 7, 7%) in traumatic amputation cases and in burns (*n* = 6, 6%).

In abdominal wall, tracheal, and penile VCAs, induction regimens varied: ALZ (*n* = 14, 14%), undefined triple therapy (*n* = 4, 4%), ATG + MMF + PDN (*n* = 1, 1%), TAC + AZP (*n* = 1, 1%), and single-drug ATG (*n* = 1, 1%). For maintenance, STR-free TAC regimens (*n* = 14, 14%) resulted in 4 AR episodes [median (IQR): 0.0 (0.0–0.8) ARs per case]. TAC + PDN + AZP (*n* = 5, 5%) resulted in 4 AR episodes [median (IQR): 0.5 (0.3–0.8) ARs per case], while TAC + MMF + PDN (*n* = 3, 3%) resulted in 1.5 AR episodes [median (IQR): 1.3–1.8]. Here, regimens were not stratified by indication due to the heterogeneity of data in the limited number of cases. Further details on immunosuppressants and rejection rates are available in Tables 2, 4.

### 3.6 Infection prophylaxis

To prevent infections in VCA recipients, prophylaxis was administered in 32% of cases (*n* = 32), including antibiotics (*n* = 28, 27%), antivirals (*n* = 25, 25%), and antifungals (*n* = 12, 12%).

Antibiotic (AB) prophylaxis most commonly included trimethoprim-sulfamethoxazole (cotrimoxazole, *n* = 19, 19%), followed by vancomycin (*n* = 7, 7%) and cefotaxime (*n* = 5, 5%). Additional antibiotics included piperacillin-tazobactam and imipenem (*n* = 2, 2% each), as well as amoxicillin-clavulanate, daptomycin, ciprofloxacin, ceftazidime, teicoplanin, ceftriaxone, and clindamycin (*n* = 1, 1% each). In 11% of cases (*n* = 11), combined AB prophylaxis was administered, with regimens including vancomycin, cefotaxime, and cotrimoxazole (*n* = 5, 5%) and other triple or dual combinations. Notably, four cases (4%) did not provide specific details about the antibiotics used. Bacterial infections occurred in 17% of cases (*n* = 17), with 8% (*n* = 8) occurring despite AB prophylaxis.

Antiviral (AV) prophylaxis primarily included valganciclovir (*n* = 13, 13%) and ganciclovir (*n* = 8, 8%), followed by acyclovir (*n* = 6, 6%), valacyclovir (*n* = 2, 2%), and cidofovir and famciclovir (*n* = 1, 1% each). Despite prophylaxis, viral infections were reported in 17% of cases (*n* = 17), with 8% (*n* = 8) occurring under AV coverage.

Antifungal (AF) prophylaxis featured micafungin (*n* = 6, 6%), nystatin (*n* = 4, 4%), fluconazole (*n* = 3, 3%), and anidulafungin (*n* = 1, 1%). Fungal infections occurred in 7% of cases (*n* = 7), with two infections (2%) reported despite AF prophylaxis. Further details on infection prophylaxis, including specific regimens and dosages, are provided in Tables 3, 4.

**Table 3:** Summary of infection prophylaxis and infectious complications.

Author	Year of publication	VCA type	Transplant indication	Length of follow-up	Infection prophylaxis	Infectious complications
Dubernard	1999	Unilateral forearm	Traumatic amputation (circular saw accident)	6 mo	AB (until POD 10)	POM 2: HSV
Schneeberger	2006	Bilateral hand	Traumatic amputation at wrist level	5 y	N/A	N/A
Bonatti	2007	Bilateral forearm	Electrical burn	24 mo	Ganciclovir, cidofovir	Alternaria alternata and CMV infection
Dubernard	2007	Face	Dog bite	18 mo	Gancyclovir (5 mg/kg BID until POD 5), valgancyclovir (900 mg/d until POM 5); trimethoprim-sulfamethoxazole (400 mg/d until POM 4), amoxicillin-clavulanate (3 g/d until POD 10)	POD 185: HSV 1, molluscum contagiosum (poxvirus)
Schneeberger	2008	Unilateral hand	Traumatic amputation	P1: 57 mo; P2: 65 mo; P3: 73 mo	N/A	N/A
Ravindra	2008	Unilateral hand	P1: firecracker accident, P2: traumatic amputation (industrial press accident)	N/A	Ganciclovir	CMV in 2 patients
Selvaggi	2009	Abdominal wall (*n* = 9), multivisceral (liver, stomach, pancreas, small bowel; *n* = 4), modified multivisceral transplants (multivisceral minus liver graft; *n* = 2); one abdomnial graft was secondary	Traumatic amputation of proximal, mid or distal forearm	P1: 19 mo; P2: 18 mo; P3: 7 mo	N/A	N/A
Schneeberger	2009	P1: unilateral hand, P2: bilateral hand, P3: bilateral hand	Intestinal motility disorders, vasculitis, trauma	N/A	N/A	CMV, varicela zoster virus
Delaere	2010	Trachea	Blunt trauma (car accident)	1 y	N/A	Bronchitis, pneumonia
Siemionow	2010	Face	Ballistic trauma	8 mo	N/A	None
Lantieri	2011	Face	P1: NF1, P2,4: burn, P3,5: ballistic trauma	38 mo	Vancomycin, cefotaxim and trimethoprim-sulfamethoxazole until POM 6; valgancyclovir (900 mg/d until POM 6)	P1: CMV, P3,4: pseudomonas, P5: HSV1
Barett	2011	Face	Electrical burn	2 y	Valganciclovir; cotrimoxazol	Candida sp. and sensitive pseudomonas aeruginosa in swab
Cavadas	2011	Bilateral forearm	Ballistic trauma	120 d	N/A	N/A
Pomahac	2011	Face	Electrical burn	1 y	N/A	None
Pei	2012	P1: right wrist, P2: right wrist, P3: right wrist, P4: right thumb, P5: double proximal forearm, P6: double wrist, P7: left proximal forearm, P8: right distral forearm, P9: double proximal forearm, P10: right proximal forearm, P11: left palm, P12: right wrist	N/A	6 w to 4 mo	N/A	P1,2: cutaneous mycosis, P3: pulmonary infection, P11: CMV
Delaere	2012	Trachea	P1: traumatic amputation, P2: explosion, P3: traumatic amputation, P4: explosion, P5: explosion;P6: thermal injury (low temperature), P7: explosion, P8: explosion, P9: machine injury, P10: machine injury, P11: machine injury, P12: traumatic amputation	P1: 10 y; P2: 2 y; P3: 1 y; P4: 1 y; P5: 9 y; P6: 8 y; P7: 7 y; P8: 2 y; P9: 6 y; P10: 1 y; P11: 2 y; P12: 2 y	N/A	N/A
Kamińska	2014	Hand	N/A	74 mo	Trimethoprim-sulfamethoxazole	2 CMV cases, tonsillitis, herpes zoster
Chandraker	2014	Face	Chemical burn (lye)	N/A	Valganciclovir (900 mg/day from POD 1 onwards); trimethoprim–sulfamethoxazole, vancomycin, imipenem–cilastin and ciprofloxacin; micafungin (until POD 10)	None
Petruzzo	2015	Bilateral hand	Pyrotechnic accident	N/A	N/A	P2: POD 152: osteitis of left ulna; P3: POD 603: asymptomatic EBV; P5: POD 81: oral cellulitis, POD 867: herpes zoster
Kanitakis	2015	P1: face, P2: bilateral hand	P1: explosion, P2: crush injury, P3: electrical burn, P4: burn injury, P5: explosion	N/A	N/A	Mollusca contagiosa, HSV1, POY 4: HPV
Diaz-Siso	2015	Bilateral upper extremity	P1: dog attack, P2: electrical burn	P1: 6 y, P2: 8.4 y	N/A	Sinusitis, femoral catheter-related stenotrophomonas maltophila bacteremia
Kim	2015	Unilateral hand	Traumatic amputation	9 mo	N/A	N/A
Kuo	2015	Unilateral hand	N/A	750 d	Ganciclovir (10 mg/kg until POD 7), valganciclovir (900 mg/d until POM 6); ceftazidime, teicoplanin, trimethoprim/sulfamethoxazole (400 mg/80 mg); nystatin (p.o.)	POW 3: fungal folliculitis, POD 140: cellulitis and axillary lymphadenopathy, POM 7: CMV
Petruzzo	2015	Face	Iatrogenic amputation due to septic shock	28 to 58 mo, median: 34 mo	N/A	HSV1; POD 185: asymptomatic EBV
Selber	2016	Face	Iatrogenic calvaria osteoradionecrosis post removal of scalp tumor	1 y	N/A	N/A
Özkan	2017	Face	N/A	1 y	Valacyclovir; sulfadoxine-pyrimethamine; nystatin (p.o.)	Complicated infectious and metabolic events
Iyer	2017	Bilateral hand	P1–2: burn injury, P3–5: ballistic trauma	11 mo to 2 y	AB, aciclovir	P1: POM 2: salmonella, POM 6: herpes labialis, POM 7: paronychia, POM 8: upper respiratory tract infection; P2: giardia lamblia
Cendales	2018	Unilateral forearm	Trauma	47 d	Valgancyclovir; trimethoprim/sulfamethoxazole (until POM 6), fluconazole (until POD 3)	N/A
Cetrulo	2018	Penis	Burn injury	24 mo	Valganciclovir, famciclovir; trimethoprim-sulfamethoxazole	N/A
Fallahian	2018	Bilateral upper extremity	N/A	3 y	N/A	N/A
Gelb	2018	Face	Subtotal penectomy due to penile cancer	6 mo	Valganciclovir (450 mg/d); trimethoprim-sulfamethoxazole (160 mg/d), piperacillin/tazobactam and clindamycin; micafungin [100 mg/d switched to fluconazole (200 mg/d) on POD 25]	None
Kwon	2018	Unilateral forearm	Traumatic amputation (meat grinder accident)	20 mo	acyclovir (250 mg), ceftriaxone (2 g), fluconazole (500 mg)	N/A
Krezdorn	2019	Face	P1–3: electrical burn, P4: chemical burn (acid), P5–6: ballistic trauma	45 to 112 mo, mean: 76,4 ± 23.5 SD	N/A	N/A
Hautz	2020	P1: bilateral distal forearm, P2: bilateral proximal forearm, P3: bilateral mid forearm, P4: unilateral wrist, P5: bilateral wrist	P1 + 3 explosion, P2: electrical burn, P4: timber machine accident, P5: car accident	P1: 19 y, P2: 16 y, P3: 13 y, P4: 7 y, P5: 5 y	N/A	P1,2,3: CMV; P2, 5: HPV; P3: myocarditis; P2: epidermatitis; P3: pangastritis
Atia	2020	Abdominal wall	Ballistic trauma	18 mo	N/A	Blood stream infection
Roy	2020	Face	Ballistic trauma	N/A	N/A	CMV, esophagitis, acute diverticulitis
Azoury	2021	Bilateral hand	Small-bowel fistulas	1 y	N/A	N/A
Govshievich	2021	Face	Tissue ischemia	1 y	Ganciclovir, valganciclovir; piperacillin-tazobactam, vancomycin, trimethoprim-sulfamethoxazole; anidulafungin	Mucormycosis
Murakami	2023	Face	Traumatic amputation	1 y	N/A	N/A
Lee	2023	Unilateral hand	Ballistic trauma	56 mo	AB	N/A
Ozmen	2023	Face	Iatrogenic amputation due to urosepsis with ARDS	48 w	Ganciclovir; daptomycin, imipenem, trimethoprim-sulfamethoxazole; fluconazole, nystatin (p.o.)	Opportunistic infections requiring hospitalization
Zaccardelli	2024	Bilateral upper extremety	Iatrogenic amputation secondary to urosepsis complicated by ARDS	12 y	N/A	HSV1, asymptomatic EBV, EBV-associated DLBCL, pneumonia, sepsis and consequent multiorgan failure

VCA, vascular composite allograft; CR, case report; CS, case series; P, patient; m, male; f, female; d, day; w, week; mo, month; y, year; NF, neurofibromatosis; POD, postoperative day; POW, postoperative week; POM, postoperative month; POY, postoperative year; BID, bidaily; AB, antibiotics; AV, antivirals; AF, antifungals; HSV, herpes simplex virus; CMV, cytomegalovirus; EBV, Epstein–Barr virus; HPV, human papillomavirus; N/A, not available.

**Table 4:** Summary of rejection episodes in various immunosuppression schemas.

Across all VCAs	Total # of cases	Total # of cases with at least one episode of AR	Total # of cases with at least one episode of CR	Total # of cases with no rejection	Percentage of cases with rejection [%]	Percentage of cases without rejection [%]	Proportion of CR out of total rejection [%]
Immunosuppression regimen alone	57	48	4	5	91.2	8.8	7.7
Unclear distinction between induction and maintenance therapy only	8	6	2	0	100	0	28.6
Induction and maintenance therapy	49	42	2	5	89.8	10.2	4.6
Immunosuppression regimen and antiinfectious regimen	36	33	0	3	91.7	8.3	0

#, number; VCA, vascular composite allograft; CR, chronic rejection; AR, acute rejection.

## 4 Discussion

Restoring form and function of amputated limbs and complex tissue defects remains a significant challenge for reconstructive surgeons. VCAs have become a valuable option for advanced tissue reconstruction (51). The aim of this systematic review is to analyze and discuss current immunosuppressive and antiinfectious strategies after VCA surgery.

### 4.1 Demographical aspects

Historically, upper extremity and face transplants are the most common types of VCAs, with well over 150 upper extremity and 40 face transplants reported globally (52). In this review, 102 VCA cases were included: 50 were UE, and 27 were face transplants.

Several factors may influence this statistic: (1) Upper extremity amputations are more frequent than severe facial trauma. Zeelenberg et al. found that upper extremity injuries in polytrauma patients are two times more likely than facial injuries (53). (2) The cost of facial VCAs is substantially higher. Siemionow et al. estimated the surgical costs of a face transplant at approximately $130,000, whereas Chung et al. reported surgical costs for a hand transplant at around $14,000 (54, 55). (3) Face transplants carry a higher risk of tissue rejection. Kollar et al. reported that ∼80% of facial VCA recipients experienced at least one episode of AR in the first postoperative year, compared to about 70% in hand transplant recipients, as shown by Petruzzo et al. (56, 57) (4) Potential donors are more willing to donate hands than faces. Sarwer et al. found that 54.6% of study participants were willing to donate hands, while only 44% were willing to donate their faces post-mortem (58). (5) Ethical concerns such as the potential loss of patient identity are more persistent in face transplants. In fact, Siemionow et al. found that facial VCA is more likely to result in identity issues compared to other types of VCA (2). However, Azher et al. concluded that facial VCA may improve body image, self-esteem and reduce chronic psychosomatic pain (59).

From a clinical perspective it is important to raise awareness and address these ethical concerns, particularly on facial VCAs, to further advance VCA procedures. For example, this could be achieved through targeted education campaigns or by informing eligible patients in trauma and rehabilitation centers about the benefits/risks and availability of VCA. Increasing awareness of facial VCA may also help improve social acceptance and reduce clinical barriers.

### 4.2 Strategies of immunosuppression

Immunosuppressive drugs are a double-edged sword. While they have revolutionized perioperative transplant care, potential side effects persist as a significant challenge (60). This trade-off underlines the importance of adequate and balanced therapeutic strategies.

ATG, TAC, MMF, and STR are key immunosuppressive components in about one-third of studies. In UE VCAs, ATG, TAC, MMF, and PDN were the most common induction combination. For maintenance, triple therapy with TAC, MMF, and PDN showed higher acute rejection (AR) rates compared to quadruple therapy, which included an additional immunosuppressant (e.g., SRL, BCL, or ERL). In fVCAs, ATG, TAC, MMF, and STR were the most common induction drugs. Maintenance therapy typically included TAC, MMF, and STR in 11% of cases, with extensions (e.g., fresh frozen plasma, bone marrow cells, BSX, extracorporeal photopheresis) reported in 19% (*n* = 8) of studies. Interestingly, AR rates were lower in non-extended triple therapy compared to extended regimens. TAC was replaced by SRL or ERL in nearly one-fourth of studies, with dosages varying widely between 4 and 7 ng/mL and 15–20 ng/mL across 15 different application schemas. No standardized immunosuppressive protocol specifically tailored for VCA was identified.

These findings echo previous research work in the field of SOT. For example, Lerut et al. found that the most frequent combination of immunosuppressants in liver transplantation involved TAC, MMF or AZP and STR, resulting in AR rates of less than 50% (61). In heart transplantation, Aliabadi et al. found that TAC-based regimens led to fewer rejections than CSA-based regimens, with AR rates of roughly 40%, as shown by Goldberg et al. (62, 63). When it comes to lung transplants, ERL and BLC were commonly administered, with a rejection rate of approximately 50% in the first postoperative year, according to Mrad et al. (64). In contrast, for kidney transplantation Kalluri et al. reported the frequent administration of TAC or CSA with adjunctive agents such as MMF and STR, resulting in an overall rejection rate of 33% to 69%, as observed by Oweira et al. (65, 66). Turning to TAC dosages, previous studies by Przepiorka et al. reported doses as high as 40 ng/mL in bone marrow transplantation (67). However, more recent studies by Kim et al. suggested lower concentrations, such as 4–7 ng/mL for liver transplantation and 10–20 ng/mL for bone marrow transplantation (67, 68). Overall, there is no clinical consensus on the ideal TAC dosage.

From a clinical point of view, immunosuppressive treatment is crucial in VCA transplantation (69). Developing standardized drug protocols could structure pharmacological therapies and improve patient outcomes. Given the limited number of VCAs, further research is needed to allow for standardized immunosuppressive drug regimens in VCA surgery.

### 4.3 Infection prophylaxis

Infectious complications in VCA recipients represent a common complication that affects up to 85% of VCA recipients as shown by Milek et al. (70). Bacterial prophylaxis was administered in nearly one-third of cases, with trimethoprim-sulfamethoxazole (cotrimoxazole) as the most common antibiotic. Despite prophylaxis, bacterial infections occurred in eight cases compared to nine without it. Antiviral prophylaxis, mainly with valganciclovir, was used in about one-fourth of cases. However, viral infections still occurred in eight cases vs. nine without prophylaxis. Fungal prophylaxis was applied in fewer than 15% of cases, primarily using micafungin and nystatin. Fungal infections were reported in two cases with prophylaxis and five without it.

Infection prophylaxis remains a significant challenge in both VCA and SOT. Bacterial prophylaxis improves outcomes, as shown by Graziano et al. (71), and reduces opportunistic infections, according to Peleg et al. (72) However, Horton et al. (73) raised concerns about side effects, such as *Clostridium difficile* infection. Abbo et al. (74) emphasized the importance of prophylaxis but highlighted risks, including MRSA colonization, surgical site infection, and dosage adequacy. Notably, prophylaxis is not standardized: cefazolin is preferred in kidney transplants, vancomycin and cefepime in heart and lung transplants, and ampicillin-sulbactam in liver and pancreas transplants.

Antiviral prophylaxis targets CMV, a major rejection risk factor. Couchoud (75) and Gupta (76) reported reduced CMV incidence and delayed onset with intravenous ganciclovir in kidney transplants. However, Elkhammas et al. (77) found no difference in CMV disease incidence, patient survival, or graft survival with AV prophylaxis. Slifkin et al. (78) noted reduced CMV morbidity but emphasized that benefits on overall survival remain unclear due to potential side effects.

Fungal prophylaxis is crucial in liver and lung transplants, given the high mortality from fungal infections. Hagerty et al. (79) reported fungal infection rates between 2% and 50%, with Kriegl et al. (80) showing reduced infection risk with antifungal (AF) prophylaxis. However, Tiew et al. (81) noted altered mycobiome diversity, which could favor resistant pathogens like mucorales. Johnson et al. (82) emphasized standardized AF approaches to identify high-risk patients and minimize side effects. Common antifungal drugs include amphotericin B and triazoles. Standardized prophylactic regimens in VCA surgery could reduce infection rates and improve outcomes. Protocols must be tailored to patient-specific characteristics to minimize complications.

Lastly, Table 4 compares immunosuppressive therapy, anti-infection prophylaxis, and their combinations. Immunosuppression alone resulted in a 91.2% rejection rate, with only 8.8% remaining rejection-free. Combined induction and maintenance therapy had an 89.8% rejection rate, with 10.2% rejection-free. Combining immunosuppression and anti-infection prophylaxis yielded a similar rejection rate (91.7%) with no chronic rejection cases. Unclear protocols or anti-infection therapy alone resulted in universal rejection (100%), highlighting the need for consistent immunosuppressive strategies.

Rejection in VCAs is difficult to assess due to their complex structure and varying immune responses across tissues. Skin, with its strong immune profile, contrasts with less immunogenic tissues like bone or muscle. Poorly vascularized areas, such as the trachea, can obscure rejection signs. Slow progression, non-specific symptoms, and a lack of standardized biomarkers complicate early detection (83). Tissue-specific characteristics, immune reactivity, prior sensitization, and comorbidities influence rejection risk. Subclinical rejection, occurring without symptoms, can lead to chronic rejection, threatening graft survival. Improved diagnostic tools, biomarkers, and tissue-specific monitoring protocols are essential. Non-invasive imaging techniques, such as PET-CT or MRI, may enable earlier detection of subtle rejection-related changes and improve patient outcomes (51).

## 5 Limitations

The findings of this analysis should be interpreted considering the following limitations: The majority of included studies are case reports or case studies, tending towards a lower LOE and NOS which might influence the reliability of results and introduce bias. Additionally, not all studies detailed the immunosuppressive regimens (e.g., missing details of therapy strategies, limited information on drug dosages). Regarding the use of infection prophylaxis, the resultant rate of 31% noted in this review could be skewed due to reporting bias, where prophylaxis was often incompletely documented or not described at all. The true rate of anti-infectious prophylaxis is possibly higher given that there are patient populations, such as older patients or those in specific geographic regions, for which prophylaxis should be utilized and tailored. For this reason, the finding of a 31% prophylaxis rate should be interpreted with caution, as it likely reflects documentation practices rather than actual clinical use. Furthermore, the heterogeneity in patient demographics as well as the varying length of follow-up may restrict the applicability and generalizability of outcomes, especially when paired with the overall small number of VCAs. Moreover, although innovative protocols such as the Pittsburgh group's bone-marrow–based approach with tacrolimus maintenance monotherapy are highly relevant, they were not included in our dataset due to overlapping patient reports across multiple publications and evolving treatment regimens, which made consistent extraction without double-counting impossible. Additionally, standardized grading criteria for VCA allograft rejection were not used across most studies included in this review. Therefore, the comparability of reported outcomes and rejection classifications remains limited. Lastly, there is no standardized protocol for immunosuppression prior, during and after VCA, deeming it difficult to compare study parameters and restricting the transferability of the results. Lastly, a regional bias is present, as several studies from Asia were excluded due to non-English publication or lack of accessible full-text, which may have further limited the global representation of VCA outcomes.

## 6 Conclusion

VCA procedures offer a novel roadmap to restore function and form. However, this roadmap is paved with significant clinical and ethical challenges. Immunosuppressive strategies are essential to ensure graft survival, yet the absence of standardized protocols causes variability in outcomes and increases patient risks. Similarly, infection prophylaxis plays a key role in VCA care, given the high susceptibility of VCA recipients to bacterial, viral and fungal infections. However, there are various schemas with different drug dosages, lacking standardization. This review underscores the need for more standardized and tailored guidelines in both immunosuppressive therapy and infection control to improve patient outcomes and graft longevity.

## Data Availability

The original contributions presented in the study are included in the article/Supplementary Material, further inquiries can be directed to the corresponding author.
