# Supplementary material for: Immunosuppressive and antiinfectious regimens in vascular composite allograft recipients—a systematic review
Source: Front Transplant. 2025 Dec 18;4:1714886. doi: 10.3389/frtra.2025.1714886 (PMC12756177; doi:10.3389/frtra.2025.1714886)
Supplement: Supplementary file 1 [file Supplementaryfile1.docx]

**12.Supplementary Digital Content**

**Supplementary Digital Content 1.**

Search strings in PubMed/MEDLINE, Web of Science, EMBASE, and CENTRAL databases with the respective number of search results.

| Database | Search String | Results |
| --- | --- | --- |
| PubMed/MEDLINE | Vascularized Composite Allotransplantation[MeSH Terms] OR  Face Transplant*[Title/Abstract] OR  Facial Transplant*[Title/Abstract] OR  Vascularized Composite Allotransplantation*[Title/Abstract] OR  Vascularized Composite Allograft*[Title/Abstract] OR  Vascularized Composite Tissue Transplantation[Title/Abstract] OR  Vascularized Allogeneic Tissue[Title/Abstract] OR  Vascularized Allograft*[Title/Abstract] OR  Composite Tissue Allotransplant*[Title/Abstract] OR  Composite Tissue Allograft*[Title/Abstract] OR  Composite Tissue Transplant*[Title/Abstract] | 2,517 |
| Web of Science | TI=(“Face Transplant*”) OR  TI=(“Facial Transplant*”) OR  TI=(“Vascularized Composite Allotransplantation*”) OR  TI=(“Vascularized Composite Allograft*”) OR  TI=(“Vascularized Composite Tissue Transplantation”) OR  TI=(“Vascularized Allogeneic Tissue”) OR  TI=(“Vascularized Allograft*”) OR  TI=(“Composite Tissue Allotransplant*”) OR  TI=(“Composite Tissue Allograft*”) OR  TI=(“Composite Tissue Transplant*”) OR  AB=(“Face Transplant*”) OR  AB=(“Facial Transplant*”) OR  AB=(“Vascularized Composite Allotransplantation*”) OR  AB=(“Vascularized Composite Allograft*”) OR  AB=(“Vascularized Composite Tissue Transplantation”) OR  AB=(“Vascularized Allogeneic Tissue”) OR  AB=(“Vascularized Allograft*”) OR  AB=(“Composite Tissue Allotransplant*”) OR  AB=(“Composite Tissue Allograft*”) OR  AB=(“Composite Tissue Transplant*”) | 2,020 |
| EMBASE | (‘Face Transplant*’):ab,ti OR  (‘Facial Transplant*’):ab,ti OR  (‘Vascularized Composite Allotransplantation*’):ab,ti OR  (‘Vascularized Composite Allograft*’):ab,ti OR  (‘Vascularized Composite Tissue Transplantation’):ab,ti OR  (‘Vascularized Allogeneic Tissue’):ab,ti OR  (‘Vascularized Allograft*’):ab,ti OR  (‘Composite Tissue Allotransplant*’):ab,ti OR  (‘Composite Tissue Allograft*’):ab,ti OR  (‘Composite Tissue Transplant*’):ab,ti | 2,531 |

**Supplemental Table 1.**

General study characteristics.

VCA=vascular composite allograft, CR=case report, CS=case series, P=patient, m=male, f=female, d=day, w=week, mo=month, y=year, NF=neurofibromatosis, ATG=antithymocyte globulin, TAC=tacrolimus, MMF=mycophenolic acid, PDN=prednisone, STR=steroids, AR=acute rejection, CR=chronic rejection, POD=postoperative day, POW=postoperative week, POM=postoperative month, POY=postoperative year, BID=bidaily, AB=antibiotics, AV=antivirals, AF=antifungals, HSV=herpes simplex virus, CMV=cytomegalovirus, EBV=Epstein-Barr virus, HPV=human papillomavirus, N/A=not available

| **Author** | **Publication year** | **Region of publication** | **Study type** | **Sample size** | **Recipient age** | **Recipient sex** | **Donor age** | **Donor sex** | **VCA type** | **Transplant indication** | **Length of follow-up** | **Rejection** | | | **Induction immunosuppression** | **Maintenance immunosuppression** | **Infection prophylaxis** |
| --- | --- | --- | --- | --- | --- | --- | --- | --- | --- | --- | --- | --- | --- | --- | --- | --- | --- |
| Atia | 2020 | USA | CR | 1 | 37 | m | 13 | m | Abdominal wall | High-output small-bowel enterocutaneous fistulas | 1 y | | AR episode | ATG (1.5 mg/kg, 4 doses) | | TAC (maintenance blood level of 15–18 ng/ml for 3 m), MMF (1,000 mg BID), PDN (20 mg/d, tapered) | N/A |
| Azoury | 2021 | USA | CR | 1 | 40 | f | N/A | N/A | Bilateral hand | Tissue ischemia | 1 y | | AR episode | ATG (75 mg, 5 doses) | | TAC, MMF (1g/d), PDN, SRL | N/A |
| Barett | 2011 | Spain | CR | 1 | 30 | m | 41 | m | Face | Ballistic trauma | 120 d | | AR: POD 3, POD 7, POD 28, POD 75, POM 3 | ATG (i.v. 2 mg/kg) 2 h pre-op, PDN (1g) | | TAC (maintenance blood level of 10-15 ng/ml), MMF (2g/d p.o.), PDN (1 mg/kg/d i.v. tapered to 10 mg/d) | Valganciclovir; cotrimoxazol |
| Bonatti | 2007 | Austria | CR | 1 | 35 | m | N/A | N/A | Bilateral forearm | Electrical burn | 24 m | | 3 AR episodes | N/A | | N/A | Ganciclovir, cidofovir |
| Cavadas | 2011 | Spain | CR | 1 | 29 | m | 25 | m | Bilateral forearm | Electrical burn | 2 y | | AR: POM 6, POM 13, POM 26 | Alemtuzumab (30 mg i.v.), PDN (POD 1: 500 mg, POD 2: 250 mg, then discontinued) | | TAC (switched to sirolimus on POD 332), MMF (2g/d) | N/A |
| Cendales | 2018 | USA | CR | 1 | 54 | m | N/A | m | Unilateral forearm | Traumatic amputation (meat grinder accident) | 20 m | | AR: POM 8 | ATG (1,5 mg/kg, 3 doses) | | Belatacept (10 mg/kg, 2 doses), TAC (5 mg/kg, maintenance blood level of 10-15 ng/ml); POM 6: TAC switched to SRL (maintenance blood level of 8-12 ng/ml), MMF (1 g BID), PDN (tapered to 10 mg/d); POM 20: belatacept 5 mg/kg monthly, MMF (500 mg BID), PDN (10 mg/d) | Valgancyclovir; trimethoprim/ sulphamethoxazole (until POM 6), fluconazole (until POD 3) |
| Cetrulo | 2018 | USA | CR | 1 | 64 | m | 27 | m | Penis | Subtotal penectomy due to penile cancer | 6 m | | AR: POD28, POD 32 | ATG, MMF, PDN | | MMF, TAC, PDN (tapered) | Valganciclovir; trimethoprim-sulfamethoxazole |
| Chandraker | 2014 | USA | CR | 1 | 45 | f | 45 | f | Face | Chemical burn (lye) | N/A | | AR: POD 12, POD 15, POD 19 | ATG 1.5 mg/kg/day for 4 days, MMF 2 g/d, STR, TAC (2mg; goal of 10 mg). POD 1: Plasmapheresis, 10 g immunoglobulin (150 mg/kg) | | TAC, MMF | Valganciclovir (900 mg/day from POD 1 onwards); trimethoprim–sulfamethoxazole, vancomycin, imipenem–cilastin and ciprofloxacin; micafungin (until POD 10) |
| Delaere | 2010 | Belgium | CR | 1 | 55 | f | N/A | m | Trachea | Blunt trauma (car accident) | 1 y | | Rejection of skin graft post discontinuation of immunosuppressive therapy | TAC (12 to 15 ng/ml), azathioprine (100 mg/day), corticosteroids (0.4 mg/kg) | | TAC (6 mg/d p.o.), azathioprine (100 mg/d), PDN (4 mg/d) | N/A |
| Delaere | 2012 | Belgium | CS | 4 | P1: 26, P2: 45, P3: 17, P4: 64 | P1: m, P2: f, P3: m, P4: m | N/A | N/A | Trachea | N/A | 6 w to 4 m | | P1: AR: POM 2, POM 4, POM 6.5, POM 7, P2: CR | Triple immunosuppression (i.v.) | | TAC (6 mg), azathioprine (100 mg), PDN (4 mg) | N/A |
| Diaz-Siso | 2015 | USA | CS | 1 | 65 | m | N/A | N/A | Bilateral upper extremity | Iatrogenic amputation due to septic shock | 28 to 58 m, median: 34 m | | AR: POM 16, POM 26 | ATG (1.5mg/kg for 4 days), PDN (500 mg/day with gradual taper, MMF (1000 mg pre-op) | | TAC (maintenance blood levels of 10–15 ng/mL for 3–21 d), MMF (1000 mg BID), PDN (20 mg on POD 5, tapered) | N/A |
| Dubernard | 1999 | France | CR | 1 | 48 | m | 41 | m | Unilateral forearm | Traumatic amputation (circular saw accident) | 6 m | | AR: POW 8–9 | ATG (75 mg/day for 10 days), TAC (blood level of 10 ng/ml to 15 ng/ml during POM 1), MMF (2 g/day), PDN (250 mg on POD 1, tapered to 20 mg/day), Daclizumab (CD25 monoclonal antibody) on POD 26 and POD 100 | | TAC (maintenance blood level of 5 ng/ml to 10 ng/ml), MMF (2 g/day), PDN (POM 3: 20 mg/day, POM 6: 15 mg/day) | AB (until POD 10) |
| Dubernard | 2007 | France | CR | 1 | 38 | f | 46 | f | Face | Dog bite | 18 m | | AR: POD 18, POD 214; POD 18: candida stomatitis due to Candida albicans | ATG for 10 days, TAC (blood concentrations between 10 to 15 ng/ml during first month), MMF (2 g/d), PDN (250 mg on day 1, 100 mg on day 2 and 60 mg/d until POD 12 with gradual taper) | | TAC (p.o.), MMF (2 g/d), PDN (10 mg/d p.o.), topical TAC and STR; POM 11: Sirolimus (maintenance blood level of 8 to 12 ng/ml), infusion of fresh frozen plasma for 4 days, high dose of i.v. immune globulin | Gancyclovir (5 mg/kg BID until POD 5), valgancyclovir (900 mg/d until POM 5); trimethoprim-sulfamethoxazole (400 mg/d until POM 4), amoxicillin-clavulanate (3 g/d until POD 10) |
| Fallahian | 2018 | USA | CR | 1 | 42 | m | Suitable match | Suitable match | Bilateral upper extremity | N/A | 3 y | | 1 AR episode | ATG (1.5 mg/kg) | | TAC (maintenance blood level of 8–10 ng/ml), MMF (720 mg BID), PDN (10 mg/d), plasmapheresis prior to discharge | N/A |
| Gelb | 2018 | USA | CR | 1 | 41 | m | 26 | m | Face | Burn injury | 24 m | | No episodes of rejection | ATG (6mg/kg, total dose of 575 mg), PDN (tapered), rituximab (1 g), TAC (5mg intra-op), MMF (1000mg) | | TAC, MMF, PDN | Valganciclovir (450 mg/d); trimethoprim-sulfamethoxazole (160 mg/d), piperacillin/tazobactam and clindamycin; micafungin (100 mg/d switched to fluconazole (200 mg/d) on POD 25) |
| Govshievich | 2021 | Canada | CR | 1 | 64 | m | Younger than recipient | m | Face | Ballistic trauma | 18 m | | AR episode | ATG, TAC (blood concentration of 10 to 15 ng/ml), MMF, PDN | | TAC (maintenance blood level of 10-15 ng/ml), MMF (same dose as in induction), PDN (tapered over 5 w) | Ganciclovir, valganciclovir; piperacillin-tazobactam, vancomycin, trimethoprim- sulfamethoxazole; anidulafungin |
| Hautz | 2020 | Austria | CS | 5 | P1: 47, P2: 41, P3: 23, P4: 55, P5: 55 | m | N/A | N/A | P1: bilateral distal forearm, P2: bilateral proximal forearm, P3: bilateral mid forearm, P4: unilateral wrist, P5: bilateral wrist | P1+3 explosion, P2: electrical burn, P4: timber machine accident, P5: car accident | P1: 19 y, P2: 16 y, P3: 13 y, P4: 7 y, P5: 5 y | | AR: all patients; in total 43 Ars; CR in POY 7 in P4 | P1, 2: ATG, P3, 4, 5: Alemtuzumab | | P1, 2, 3, 5: TAC, MMF, STR; P4: TAC, MMF; belatacept in P5 (discontinued after 3 y in P2 and P3) | N/A |
| Iyer | 2017 | India | CS | 2 | N/A | N/A | N/A | N/A | Bilateral hand | N/A | 1 y | | P1: POW2, POW4, POM 8, POM 9; P2: POM 1 | ATG (1.5 mg/kg i.v.), TAC (0.05 mg/kg), PDN (500 mg i.v.), MMF (1000 mg); POD 0: ATG (1.5 mg/kg), TAC (0.1 mg/kg BID), PDN (250 mg i.v.), MMF (1000 mg BID); POD 1-5: ATG (1.5 mg/kg for 3 days), TAC (0.1 mg/kg BID), PDN (0.5 mg/kg/day p.o.), MMF (1000 mg BID) | | TAC, MMF (1000 mg BID), PDN (0.5 mg/kg/d) | AB, aciclovir |
| Kamińska | 2014 | Poland | CS | 5 | P1: 56, P2: 28, P3: 34, P4: 29, P5: 30 | P1: f, P2: m, P3: m, P4: m, P5: m | P1: 47, P2: 50, P3: 41, P4: 53, P5: 51 | P1: f, P2: m, P3: m, P4: f, P5: f | Hand | N/A | 74 m | | AR in P1: 1; P2: 2; P3: 2; P4: 7; P5: 2 | Basiliximab | | TAC (maintenance blood levels of 10-15 ng/ml), MMF (2 g/d), PDN (20-40 mg/d) | Trimethoprim-sulfamethoxazole |
| Kanitakis | 2015 | France | CS | 2 | P1: 38, P2: 27 | f | P1: 46, P2: 40 | f | P1: face, P2: bilateral hand | P1: dog attack, P2: electrical burn | P1: 6 y, P2: 8.4 y | | P1: no rejection episodes; P2: several AR episodes | P1: TAC, MMF, PDN, ATG, POD 4 and 11: bone marrow infusion, P2: N/A | | P1: sirolimus (from POM 11 onwards), MMF, PDN, extracorporeal photochemotherapy over 2 years; P2: TAC, MMF, STR | N/A |
| Kim | 2015 | USA | CR | 1 | 27 | f | N/A | N/A | Unilateral hand | N/A | 750 d | | AR: POD 717 | TAC, MMF, ATG, Everolimus, PDN | | TAC, MMF, ATG, Everolimus, PDN | N/A |
| Krezdorn | 2019 | USA | CS | 6 | P1: 25, P2: 59, P3: 30, P4: 44, P5: 39, P6: 33 | P1: m, P2: m, P3: m, P4: m, P5: f, P6: m | P1: 48, P2: 60, P3: 31, P4: 56, P5: 23, P6: 51 | Suitable match | Face | P1-3: electrical burn, P4: chemical burn (acid), P5-6: ballistic trauma | 45 to 112 m, mean: 76,4 ±23.5 SD | | Number of AR: P1: 5, P2: 4, P3: 5, P4: 6, P5: 2, P6: 5; CR in P1: POM 25, P2: POM 68 | P1,2,3,4: TAC, MMF, PDN, P5: TAC, MMF, P6: TAC, MMF, PDN, Belatacept | | N/A | N/A |
| Kuo | 2015 | Taiwan | CR | 1 | 45 | m | 37 | m | Unilateral hand | Traumatic amputation | 9 m | | AR: POM 3.5, POD 105 | ATG (1.25 mg/kg for 10 days), PDN (pre-op: 500 mg, POD 0: 250 mg, POD 1: 125 mg, tapered to 10 mg); POD 1: TAC (blood concentrations of 10–15 ng/mL until POM 6, 8 to 10 ng/mL after 6 months, and then maintaining serum levels of 5–8 ng/mL). | | TAC, MMF (2 g/d), PDN (10 mg/d) | Ganciclovir (10 mg/kg until POD 7), valganciclovir (900 mg/d until POM 6); ceftazidime, teicoplanin, trimethoprim/sulfamethoxazole (400 mg/80 mg); nystatin (p.o.) |
| Kwon | 2018 | Korea | CR | 1 | 35 | m | 49 | m | Unilateral forearm | Trauma | 47 d | | AR: POD 6, POD 47 | Basiliximab (IL-2 receptor blocker, 20 mg) | | TAC (5 mg/d), MMF (750 mg/d), PDN (125 mg/d) | acyclovir (250 mg), ceftriaxone (2 g), fluconazole (500 mg) |
| Lantieri | 2011 | France | Prospective cohort study | 5 | P1: 29, P2: 39, P3: 27, P4: 37, P5: 33 | m | P1: 65, P2: N/A, P3: 43, P4: 59, P5: 55 | m | Face | P1: NF1, P2,4: burn, P3,5: ballistic trauma | 38 m | | P1: AR: POD 28, POD 64; P2: N/A; P3: AR: POD 0; P4: No rejection episode; P5: AR: POD 5 | ATG (1 mg/kg/day for 10 days), TAC (blood concentration of 10-13 ng/mL in POM 3), MMF (2 g/day), PDN (500 mg on day 1, 250 mg on day 2, 120 mg on day 3, 60 mg/day for 7 days, tapered to 10 mg/day) | | TAC (maintenance blood level of 8-10 ng/ml), MMF, PDN; P3, 4, 5: extracorporeal photopheresis (BIW for 1 m, MIW for 3 m) | Vancomycin, cefotaxim and trimethoprim-sulfamethoxazole until POM 6; valgancyclovir (900 mg/d until POM 6) |
| Lee | 2023 | South Korea | CR | 1 | 62 | m | N/A | N/A | Unilateral hand | Traumatic amputation | 1 y | | AR: POD 33, POD 41 | TAC (3mg pre-op then 4 mg/d, blood concentration of 6–8 ng/ml), STR, basiliximab (20 mg BID pre-op and POD 4), PDN (100 mg intra-op); "standard immunosuppressive regimen for kidney transplantation" | | TAC (maintenance level of 6-8 ng/ml), MMF (started on POD 14 at 1,000 mg/d), PDN (tapered to 10 mg/d by POD 17) "standard immunosuppressive regimen for kidney transplantation" | AB |
| Murakami | 2023 | USA | CS | 2 | P1: 57, P2: 60 | P1: f, P2: m | N/A | N/A | Face | Animal attack | 48 w | | P1: AR: POM 2, POM 17, POM 30, POM 47, POM 58 | P1: ATG (POM 6: blood concentration of 6-8 ng/ml), MMF (1.500 mg/d), PDN (5 mg/d), started on "IL-2 protocol" | | P1: TAC, MMF, PDN (discontinued at POM 4.5; P2: POM 6: TAC (maintenance blood level of 6-8 ng/ml), MMF (1,500 mg/d), PDN (5 mg/d), started on the "IL-2 protocol". P2: 30% of the IL-2 dose from P1 | N/A |
| Özkan | 2017 | Turkey | CS | 5 | P1: 19, P2: 35, P3: 26, P4: 54, P5: 22 | m | P1: 37, P2: 19, P3: 42, P4: 31, P5: 34 | m | Face | P1-2: burn injury, P3-5: ballistic trauma | 11 m to 2 y | | AR: all patients between POM 6 and POY 5; P1: 12 AR in POM 12; P2: POY 1; P3: POM 15; P4: POM 24 | ATG (2.5 mg/kg/day intra-op), PDN (1000 mg, tapered to 20 mg in POW 1); POD 4: TAC (0.2 mg/kg/day, blood concentration of 15 to 20 ng/ml); ATG stopped on POD 7–10 | | P1, 2, 3: TAC (maintenance blood levels of 15-20 ng/ml until POM 3, 7–10 ng/ml until POM 6), MMF (2 g/d), PDN (20 mg/d, tapered to 10 mg/d by POM 6); P4: modified protocol | Valacyclovir; sulfadoxine-pyrimethamine; nystatin (p.o.) |
| Ozmen | 2023 | Turkey | CR | 1 | 20 | f | 29 | f | Face | Ballistic trauma | 56 m | | AR: POD 26 | PDN (1000 mg), ATG (100 mg), TAC (5 mg BID), MMF (1000 mg BID) | | TAC (2 doses at 8 mg), azathioprine (2 doses at 50 mg), PDN (40 mg/morning, 20 mg/evening) | Ganciclovir; daptomycin, imipenem, trimethoprim-sulfamethoxazole; fluconazole, nystatin (p.o.) |
| Pei | 2012 | China | Retrospective cohort study | 12 | P1: 39, P2: 27, P3: 25, P4: 24, P5: 37, P6: 19, P7: 50, P8: 43, P9: 52, P10: 37, P11: 19, P12: 38 | m | P1: 29, P2: 25, P3: 30, P4: 29, P5: 35, P6: 20, P7: 48, P8: 35, P9: 50, P10: 42, P11: 24, P12: 23 | P1: m, P2: m, P3: m, P4: m, P5: m, P6: m, P7: m, P8: m, P9: m, P10: m, P11: m, P12: m | P1: right wrist, P2: right wrist, P3: right wrist, P4: right thumb, P5: double proximal forearm, P6: double wrist, P7: left proximal forearm, P8: right distral forearm, P9: double proximal forearm, P10: right proximal forearm, P11: left palm, P12: right wrist | P1: traumatic amputation, P2: explosion, P3: traumatic amputation, P4: explosion, P5: explosion;P6: thermal injury (low temperature), P7: explosion, P8: explosion, P9: machine injury, P10: machine injury, P11: machine injury, P12: traumatic amputation | P1: 10 y; P2: 2 y; P3: 1 y; P4: 1 y; P5: 9 y; P6: 8 y; P7: 7 y; P8: 2 y; P9: 6 y; P10: 1 y; P11: 2 y; P12: 2 y | | P1: AR every POY; P2: AR: POM 15; P3: AR once post-op; P4: 1 AR; P5: AR every POY; P6: AR every POY; P7: AR every POY; P8: AR: POM 6, POY 2; P9: AR: POY 1, POY 3, POY 5, POY 6; P10: POM 7; P11: POW 4, POW 8, POY 2; P12: POY 2 | P1: ATG (100 mg/d), TAC (5 mg/d), MMF (750 mg/d) PDN (1 g/d), P2: ATG (100 mg/d) TAC (5 mg/d), MMF (750 mg/d), PDN (1 g/d), P3: CTX (400 mg/d), P4: CTX (400 mg/d), P5: ATG (100 mg/d), TAC (5 mg/d), MMF (500 mg/d), PDN (1 g/d), P6: ATG (80 mg/d), TAC (5 ng/ml), PDN (800 mg/d), P7: ATG (80 mg/d), TAC (5 ng/ml), PDN (800 mg/d), P8: ATG (80 mg/d), TAC (5 ng/ml), PDN (800 mg/d), P9: ATG (80 mg/d), TAC (5 ng/ml), PDN (800 mg/d), P10: N/A, P11: N/A, P12: N/A | | P1: TAC (3 mg/d), MMF (stopped after 6 m), PDN (5 mg/d); P2: N/A; P3: TAC (1 mg/d), MMF (1 g/d), PDN (10 mg/d); P4: TAC (1 mg/d), MMF (1 g/d), PDN (10 mg/d), P5: TAC, MMF, PDN, P6: TAC (3 mg/d), MMF (stopped after 6 m), PDN (5 mg/d); P7: TAC (3 mg/d), MMF (stopped after 6 m), PDN (5 mg/d); P8: TAC (3 mg/d), MMF (stopped after 6 m), PDN (5 mg/d); P9: TAC (3 mg/d), MMF (stopped after 6 m), PDN (5 mg/d); P10: N/A; P11: N/A; P12: N/A | N/A |
| Petruzzo | 2015 | France | CR | 1 | 27 | m | N/A | N/A | Face | Pyrotechnic accident | N/A | | AR: POD 41, POD 103, POD 186, POD 239, POD 474, POD 527, POD 540, POD 931 | ATG | | TAC (maintenance blood levels of 5-10 ng/ml), MMF (2 g/d); POD 4: PDN (5 mg/d), infusion of donor bone-marrow cells; At present: everolimus (3 mg/d), STR (16 mg/d), extracorporal photochemotherapy | N/A |
| Petruzzo | 2015 | France | CS | 5 | P1: 33, P2: 21, P3: 27, P4: 29, P5: 21 | P1: m, P2: m, P3: f, P4: m, P5: m | P1: 18, P2: 45, P3: 40, P4: 29, P5: 18 | N/A | Bilateral hand | P1: explosion, P2: crush injury, P3: electrical burn, P4: burn injury, P5: explosion | N/A | | P1: AR: POD 53, POD 72; P2: POD 57, POD 86, POD 2759; P3: POD 16, POD 271, POD 635, POD 951, POD 1365, POD 1855; P4: POD 65; P5: POD 10, POD 350, POD 560 | P1: ATG 1.25 mg/kg/d for 10 days, P2-5: ATG (3 mg/kg on POD 1 and 2 mg/kg on POD 2); PDN 250 mg on POD 1 (tapered: 20 mg/d), TAC (0.1 mg/kg since POD 2 (blood level of 10 and 15 ng/mL) and MMF (2 g/d) | | P1, 2, 4, 5: TAC (maintenance blood level of 5-10 ng/ml tapered to 5-8 ng/ml), PDN (5 mg/d), MMF (2 g/d), P3: TAC (maintenance blood level of 4-7 ng/ml), PDN (5 mg/d), sirolimus (maintenance blood level of 6-10 ng/ml), MMF (1 g/d); POM 26: P5 switched from TAC to sirolimus for 14 m | N/A |
| Pomahac | 2011 | USA | CR | 1 | 59 | m | 60 | m | Face | Electrical burn | 1 y | | AR: POD 17, POD 74, POD 107 | ATG (1.5 mg/kg), MMF (1000 mg) pre-op, PDN (500 mg) intra-op | | TAC (maintenance blood level of 10–15 ng/ml), MMF (2 g/d), PDN (tapered to 20 mg by POD 5); POD 106: TAC (5 mg BID), MMF (720 mg), PDN (10 mg); POM 9: TAC (1 mg BID, maintenance blood level of 5-8 ng/ml), POD 360: PDN discontinued | N/A |
| Ravindra | 2008 | USA | CS | 2 | P1: 37, P2: 54 | m | N/A | N/A | Unilateral hand | P1: firecracker accident, P2: traumatic amputation (industrial press accident) | N/A | | P1: AR: POM 2, POM 5, POM 7; P2: AR | P1: 20 mg of basiliximab pre-op and on POD 4, P2: 30 mg alemtuzumab intra-op | | P1: TAC (maintenance blood level of 15-20 ng/mL until POM 6), MMF (1 g BID), PDN (tapered to 7.5 mg/d by POM 6; POY 8: TAC (maintenance blood level of 6-8 ng/ml), MMF (1 g BID), PDN (7.5 mg/d), P3: TAC (maintenance blood level of 10-15 ng/ml until POM 6, then 8-10 ng/ml), MMF, PDN (3 doses peri-op until POD 3) | Ganciclovir |
| Roy | 2020 | Canada | CR | 1 | 65 | m | N/A | N/A | Face | Ballistic trauma | N/A | | AR: POD 50, POD 56, POD 286 | ATG (10mg/kg i.v.), TAC, MMF (2g/d), PDN (50mg/d i.v., tapered to 25mg) | | TAC (maintenance blood level of 10-15 ng/ml until POM 6, then 8 ng/ml), MMF (1g i.v. BID), PDN (50 mg/d i.v., tapered to 25mg/d), POW 10: topical TAC added; basiliximab on POD 105, 133, 161 and 217 | N/A |
| Schneeberger | 2006 | Austria | CR | 1 | 48 | m | N/A | N/A | Bilateral hand | Traumatic amputation at wrist level | 5 y | | AR: POD 55, POD 188, POM 48 | ATG | | TAC (maintenance blood level of 10ng/ml), MMF (2g/d), PDN (5 mg), POM 30: SRL (4-8 ng/ml), TAC (3-4 ng/mL), POM 36: STR weaned, POM 39: TAC discontinued, leaving patient on SRL and MMF | N/A |
| Schneeberger | 2008 | USA | CS | 3 | P1: 22, P2: 32, P3: 36 | m | N/A | N/A | Unilateral hand | Traumatic amputation | P1: 57 m; P2: 65 m; P3: 73 m | | P1: AR: POM 43 months PO; P2: AR: POM 3, POM 27; P3: POD 10, POD 21, POD 50, POD 77 | P1: ATG, P2: Basiliximab, P3: Basiliximab | | P1: TAC, MMF, STR, P2: TAC, MMF, STR, P3: SRL, MMF, steroids | N/A |
| Schneeberger | 2009 | USA | CS | 3 | P1: 54, P2: 46, P3: 31 | P1: male, P2: female, P3: male | N/A | N/A | P1: unilateral hand, P2: bilateral hand, P3: bilateral hand | Traumatic amputation of proximal, mid or distal forearm | P1: 19 m; P2: 18 m; P3: 7 m | | P1: AR: XXXX POM 18; P2: AR: POD 120, POD 221; P3: AR: POD 30, POD 170 | TAC (5 mg p.o.), MMF (2 g p.o.), basiliximab (1 amp. i.v.) and PDN (1 g i.v.). POD 1: TAC (5 mg p.o. BID), MMF (2g/d), MPDN (0.5 g intravenously).POD 2: TAC (5 mg p.o. BID), MMF (2 g/day), POD 3 to 7 TAC adjusted to 20 ng/mL | | P1: TAC (maintenance blood level of 10-15 ng/ml until POM 6, then 5-10 ng/ml) MMF (p.o.); POY 1: TAC (2 mg BID), MMF (500mg BID); P2: TAC (switched to sirolimus on POD 190), MMF, STR; P3: TAC, MMF, STR | N/A |
| Selber | 2016 | USA | CR | 1 | 55 | m | 33 | m | Face | Iatrogenic calvaria osteoradionecrosis post removal of scalp tumor | 1 y | | AR: POW 11 | ATG for (7.14mg/kg cumulative dose), PDN (1500mg i.v.) | | TAC (maintenance blood level of 7-10ng/ml), MMF (1g BID), PDN (5mg/d p.o.) daily, topical TAC | N/A |
| Selvaggi | 2009 | USA | CS | 14 | 10 adult, 4 pediatric | N/A | N/A | N/A | Abdominal wall (n=9), multivisceral (liver, stomach, pancreas, small bowel; n= 4), modified multivisceral transplants (multivisceral minus liver graft; n=2); one abdomnial graft was secondary | Gardner syndrome (n=5), trauma (n=3), intestinal motility disorders (n=3), gastroschisis (n=2) and Churg-Strauss vasculitis (n=1) | N/A | | POD 1 and 6: graft losses to vascular thrombosis; AR: 4 episodes resolved | Alemtuzumab | | STR-free TAC based maintenance | N/A |
| Siemionow | 2010 | USA | CR | 1 | 45 | f | N/A | N/A | Face | Ballistic trauma | 8 m | | AR | ATG | | TAC, MMF, STR | N/A |
| Zaccardelli | 2024 | USA | CR | 1 | 65 | m | N/A | N/A | Bilateral upper extremety | Iatrogenic amputation secondary to urosepsis complicated by ARDS | 12 y | | AR: POM 26, POM 37 | ATG (4 doses i.v.) | | TAC (maintenance blood level of 10–15 ng/ml), MMF (1,000 mg BID), PDN (7.5 mg/D); TAC and MMF weaned to 5 ng/ml and 360 mg/d | N/A |

**Supplemental table 2.**

Study rating based on level of evidence.

CR=case report, CS=case series, DOI=digital object identifier, CEBM=Center for Evidence Based Medicine

| **Author** | **Title** | **DOI** | **Study type** | **Sample size** | **Level of evidence (CEBM-criteria)** | |
| --- | --- | --- | --- | --- | --- | --- |
| Dubernard | Human hand allograft: Report on first 6 months | DOI: 10.1016/s0140-6736(99)02062-0 | CR | 1 | | Level 4 |
| Schneeberger | Status 5 Years after Bilateral Hand Transplantation | DOI: 10.1111/j.1600-6143.2006.01266.x | CR | 1 | | Level 4 |
| Bonatti | Soft Tissue Infection in a Forearm Transplant Recipient | DOI: 10.1089/sur.2006.095 | CR | 1 | | Level 4 |
| Dubernard | Outcomes 18 Months after the First Human Partial Face Transplantation | DOI: 10.1056/NEJMoa072828 | CR | 1 | | Level 4 |
| Schneeberger | Atypical Acute Rejection After Hand Transplantation | DOI: 10.1111/j.1600-6143.2007.02105.x | CS | 3 | | Level 4 |
| Ravindra | Hand transplantation in the United States: Experience with 3 patients | DOI: 10.1016/j.surg.2008.06.025 | CS | 2 | | Level 4 |
| Schneeberger | Alemtuzumab: Key for Minimization of Maintenance Immunosuppression in Reconstructive Transplantation? | DOI: 10.1016/j.transproceed.2009.01.018 | CS | 3 | | Level 4 |
| Selvaggi | Abdominal Wall Transplantation: Surgical and Immunologic Aspect | DOI: 10.1016/j.transproceed.2009.01.020 | CS | 14 | | Level 4 |
| Delaere | Tracheal Allotransplantation after Withdrawal of Immunosuppressive Therapy | DOI: 10.1056/NEJMoa0810653 | CR | 1 | | Level 4 |
| Siemionow | First U.S. Near-Total Human Face Transplantation: A Paradigm Shift for Massive Complex Injuries | DOI: 10.1097/PRS.0b013e3181c15c4c | CR | 1 | | Level 4 |
| Lantieri | Feasibility, Reproducibility, Risks and Benefits of Face Transplantation: A Prospective Study of Outcomes | DOI: 10.1111/j.1600-6143.2010.03406.x | Prospective cohort study | 5 | | Level 3 |
| Cavadas | Bilateral Trans-humeral Arm Transplantation: Result at 2 years | DOI: 10.1111/j.1600-6143.2011.03503.x | CR | 1 | | Level 4 |
| Barett | Full Face Transplant: The First Case Report | DOI: 10.1097/SLA.0b013e318226a607 | CR | 1 | | Level 4 |
| Pomahac | Restoration of Facial Form and Function After Severe Disfigurement from Burn Injury by a Composite Facial Allograft | DOI: 10.1111/j.1600-6143.2010.03368.x | CR | 1 | | Level 4 |
| Delaere | Learning Curve in Tracheal Allotransplantation | DOI: 10.1111/j.1600-6143.2012.04125.x | CS | 4 | | Level 4 |
| Pei | A Report of 15 Hand Allotransplantations in 12 Patients and Their Outcomes in China | DOI: 10.1097/TP.0b013e31826c3915 | Retrospective cohort study | 12 | | Level 3 |
| Kamińska | Significant Infections After Hand Transplantation in a Polish Population | DOI: 10.1016/j.transproceed.2014.08.028 | CS | 5 | | Level 4 |
| Chandraker | The Management of Antibody-Mediated Rejection in the First Presensitized Recipient of a Full-Face Allotransplant | DOI: 10.1111/ajt.12715 | CR | 1 | | Level 4 |
| Petruzzo | Clinicopathological Findings of Chronic Rejection in a Face Grafted Patient | DOI: 10.1097/TP.0000000000000765 | CR | 1 | | Level 4 |
| Petruzzo | Outcomes After Bilateral Hand Allotransplantation: A Risk/Benefit Ratio Analysis | DOI: 10.1097/SLA.0000000000000627 | CS | 5 | | Level 4 |
| Kanitakis | "Premalignant and Malignant Skin Lesions in Two Recipients of Vascularized Composite Tissue Allografts (Face, Hands)" | DOI: 10.1155/2015/356459 | CS | 2 | | Level 4 |
| Kuo | The First Hand Allotransplantation in Taiwan: A Report at 9 Months | DOI: 10.1097/SAP.0000000000000758 | CR | 1 | | Level 4 |
| Kim | Clonal CD8+ T-Cell Persistence and Variable Gene Usage Bias in a Human Transplanted Hand | DOI: 10.1371/journal.pone.0136235 | CR | 1 | | Level 4 |
| Diaz-Siso | "Initial Experience of Dual Maintenance: Immunosuppression With Steroid Withdrawal in Vascular Composite Tissue Allotransplantation" | DOI: 10.1111/ajt.13103 | CR | 1 | | Level 4 |
| Selber | Simultaneous Scalp, Skull, Kidney and Pancreas Transplant from a Single Donor | DOI: 10.1097/PRS.0000000000002153 | CR | 1 | | Level 4 |
| Iyer | First two bilateral hand transplantations in India: Immediate post‑operative care, immunosuppression protocol and monitoring | DOI: 10.4103/ijps.IJPS_96_17 | CS | 2 | | Level 4 |
| Özkan | Face allotransplantation for various types of facial disfigurements: A series of five cases | DOI: 10.1002/micr.30272 | CS | 5 | | Level 4 |
| Kwon | Anesthetic management of the first forearm transplantation in Korea | DOI: 10.4097/kjae.2018.71.1.66 | CR | 1 | | Level 4 |
| Gelb | Absence of Rejection in a Facial Allograft: Recipient with a Positive Flow Crossmatch 24 Months after Induction with Rabbit Anti-Thymocyte Globulin and Anti-CD20 Monoclonal Antibody | DOI: 10.1155/2018/7691072 | CR | 1 | | Level 4 |
| Fallahian | Eponychial lesions following bilateral upper extremity vascular composite allotransplantation: A case report | DOI: 10.1080/23320885.2018.1431047 | CR | 1 | | Level 4 |
| Cetrulo | Penis Transplantation: First US Experience | DOI: 10.1097/SLA.0000000000002241 | CR | 1 | | Level 4 |
| Cendales | De novo belatacept in clinical vascularized composite allotransplantation | DOI: 10.1111/ajt.14910 | CR | 1 | | Level 4 |
| Krezdorn | Chronic Rejection of Human Face Allografts | DOI: 10.1111/ajt.15143 | CS | 6 | | Level 4 |
| Hautz | Long-term outcome after hand and forearm transplantation – a retrospective study | DOI: 10.1111/tri.13752 | CS | 5 | | Level 4 |
| Govshievich | Face Transplant: Current Update and First Canadian Experience | DOI: 10.1097/PRS.0000000000007890 | CR | 1 | | Level 4 |
| Roy | Lymphocytic Vasculitis Associated With Mild Rejection in a Vascularized Composite Allograft Recipient: A Clinicopathological Study | DOI: 10.1097/TP.0000000000003241 | CR | 1 | | Level 4 |
| Atia | Synchronous Abdominal Wall and Small-bowel Transplantation: A 1-year Follow-up | DOI: 10.1097/GOX.0000000000002995 | CR | 1 | | Level 4 |
| Azoury | Successful transatlantic bilateral hand transplant in a young female highly sensitized to HLA class II antigens | DOI: 10.1016/j.trim.2021.101377 | CR | 1 | | Level 4 |
| Lee | One Year Experience of the Hand Allotransplantation First Performed after Korea Organ Transplantation Act Amendment | DOI: 10.1055/a-2059-5570. | CR | 1 | | Level 4 |
| Ozmen | First Composite Woman-to-Woman Facial Transplantation in Turkey: Challenges and Lessons to Be Learned | DOI: 10.1097/SAP.0000000000003323 | CR | 1 | | Level 4 |
| Murakami | Low-dose interleukin-2 promotes immune regulation in face transplantation: A pilot study | DOI: 10.1016/j.ajt.2023.01.016 | CS | 2 | | Level 4 |
| Zaccardelli | Case Report: Post-transplant lymphoproliferative disorder as a serious complication of vascularized composite allotransplantation | DOI: 10.3389/frtra.2024.1339898 | CR | 1 | | Level 4 |

**Supplemental Table 3.**

Study rating based on the Newcastle-Ottawa-scale, DOI=digital object identifier

| **Author** | **Title** | **DOI** | **Selection of study cohort** | **Comparability of cohorts** | **Assessment of outcomes** | **Final score** |
| --- | --- | --- | --- | --- | --- | --- |
| Dubernard | Human hand allograft: Report on first 6 months | DOI: 10.1016/s0140-6736(99)02062-0 | 2 | 0 | 2 | 4 |
| Schneeberger | Status 5 Years after Bilateral Hand Transplantation | DOI: 10.1111/j.1600-6143.2006.01266.x | 2 | 0 | 2 | 4 |
| Bonatti | Soft Tissue Infection in a Forearm Transplant Recipient | DOI: 10.1089/sur.2006.095 | 2 | 0 | 2 | 4 |
| Dubernard | Outcomes 18 Months after the First Human Partial Face Transplantation | DOI: 10.1056/NEJMoa072828 | 2 | 0 | 2 | 4 |
| Schneeberger | Atypical Acute Rejection After Hand Transplantation | DOI: 10.1111/j.1600-6143.2007.02105.x | 2 | 1 | 2 | 5 |
| Ravindra | Hand transplantation in the United States: Experience with 3 patients | DOI: 10.1016/j.surg.2008.06.025 | 2 | 1 | 2 | 5 |
| Schneeberger | Alemtuzumab: Key for Minimization of Maintenance Immunosuppression in Reconstructive Transplantation? | DOI: 10.1016/j.transproceed.2009.01.018 | 2 | 1 | 2 | 5 |
| Selvaggi | Abdominal Wall Transplantation: Surgical and Immunologic Aspect | DOI: 10.1016/j.transproceed.2009.01.020 | 2 | 1 | 2 | 5 |
| Delaere | Tracheal Allotransplantation after Withdrawal of Immunosuppressive Therapy | DOI: 10.1056/NEJMoa0810653 | 2 | 0 | 2 | 4 |
| Siemionow | First U.S. Near-Total Human Face Transplantation: A Paradigm Shift for Massive Complex Injuries | DOI: 10.1097/PRS.0b013e3181c15c4c | 2 | 0 | 2 | 4 |
| Lantieri | Feasibility, Reproducibility, Risks and Benefits of Face Transplantation: A Prospective Study of Outcomes | DOI: 10.1111/j.1600-6143.2010.03406.x | 2 | 1 | 2 | 5 |
| Cavadas | Bilateral Trans-humeral Arm Transplantation: Result at 2 years | DOI: 10.1111/j.1600-6143.2011.03503.x | 2 | 0 | 2 | 4 |
| Barett | Full Face Transplant: The First Case Report | DOI: 10.1097/SLA.0b013e318226a607 | 2 | 0 | 2 | 4 |
| Pomahac | Restoration of Facial Form and Function After Severe Disfigurement from Burn Injury by a Composite Facial Allograft | DOI: 10.1111/j.1600-6143.2010.03368.x | 2 | 0 | 2 | 4 |
| Delaere | Learning Curve in Tracheal Allotransplantation | DOI: 10.1111/j.1600-6143.2012.04125.x | 2 | 1 | 2 | 5 |
| Pei | A Report of 15 Hand Allotransplantations in 12 Patients and Their Outcomes in China | DOI: 10.1097/TP.0b013e31826c3915 | 2 | 1 | 2 | 5 |
| Kamińska | Significant Infections After Hand Transplantation in a Polish Population | DOI: 10.1016/j.transproceed.2014.08.028 | 2 | 1 | 2 | 5 |
| Chandraker | The Management of Antibody-Mediated Rejection in the First Presensitized Recipient of a Full-Face Allotransplant | DOI: 10.1111/ajt.12715 | 2 | 0 | 2 | 4 |
| Petruzzo | Clinicopathological Findings of Chronic Rejection in a Face Grafted Patient | DOI: 10.1097/TP.0000000000000765 | 2 | 0 | 2 | 4 |
| Petruzzo | Outcomes After Bilateral Hand Allotransplantation: A Risk/Benefit Ratio Analysis | DOI: 10.1097/SLA.0000000000000627 | 2 | 1 | 2 | 5 |
| Kanitakis | "Premalignant and Malignant Skin Lesions in Two Recipients of Vascularized Composite Tissue Allografts (Face, Hands)" | DOI: 10.1155/2015/356459 | 2 | 1 | 2 | 5 |
| Kuo | The First Hand Allotransplantation in Taiwan: A Report at 9 Months | DOI: 10.1097/SAP.0000000000000758 | 2 | 0 | 2 | 4 |
| Kim | Clonal CD8+ T-Cell Persistence and Variable Gene Usage Bias in a Human Transplanted Hand | DOI: 10.1371/journal.pone.0136235 | 2 | 0 | 2 | 4 |
| Diaz-Siso | "Initial Experience of Dual Maintenance: Immunosuppression With Steroid Withdrawal in Vascular Composite Tissue Allotransplantation" | DOI: 10.1111/ajt.13103 | 2 | 0 | 2 | 4 |
| Selber | Simultaneous Scalp, Skull, Kidney and Pancreas Transplant from a Single Donor | DOI: 10.1097/PRS.0000000000002153 | 2 | 0 | 2 | 4 |
| Iyer | First two bilateral hand transplantations in India: Immediate post‑operative care, immunosuppression protocol and monitoring | DOI: 10.4103/ijps.IJPS_96_17 | 2 | 1 | 2 | 5 |
| Özkan | Face allotransplantation for various types of facial disfigurements: A series of five cases | DOI: 10.1002/micr.30272 | 2 | 1 | 2 | 5 |
| Kwon | Anesthetic management of the first forearm transplantation in Korea | DOI: 10.4097/kjae.2018.71.1.66 | 2 | 0 | 2 | 4 |
| Gelb | Absence of Rejection in a Facial Allograft: Recipient with a Positive Flow Crossmatch 24 Months after Induction with Rabbit Anti-Thymocyte Globulin and Anti-CD20 Monoclonal Antibody | DOI: 10.1155/2018/7691072 | 2 | 0 | 2 | 4 |
| Fallahian | Eponychial lesions following bilateral upper extremity vascular composite allotransplantation: A case report | DOI: 10.1080/23320885.2018.1431047 | 2 | 0 | 2 | 4 |
| Cetrulo | Penis Transplantation: First US Experience | DOI: 10.1097/SLA.0000000000002241 | 2 | 0 | 2 | 4 |
| Cendales | De novo belatacept in clinical vascularized composite allotransplantation | DOI: 10.1111/ajt.14910 | 2 | 0 | 2 | 4 |
| Krezdorn | Chronic Rejection of Human Face Allografts | DOI: 10.1111/ajt.15143 | 2 | 1 | 2 | 5 |
| Hautz | Long-term outcome after hand and forearm transplantation – a retrospective study | DOI: 10.1111/tri.13752 | 2 | 1 | 2 | 5 |
| Govshievich | Face Transplant: Current Update and First Canadian Experience | DOI: 10.1097/PRS.0000000000007890 | 2 | 0 | 2 | 4 |
| Roy | Lymphocytic Vasculitis Associated With Mild Rejection in a Vascularized Composite Allograft Recipient: A Clinicopathological Study | DOI: 10.1097/TP.0000000000003241 | 2 | 0 | 2 | 4 |
| Atia | Synchronous Abdominal Wall and Small-bowel Transplantation: A 1-year Follow-up | DOI: 10.1097/GOX.0000000000002995 | 2 | 0 | 2 | 4 |
| Azoury | Successful transatlantic bilateral hand transplant in a young female highly sensitized to HLA class II antigens | DOI: 10.1016/j.trim.2021.101377 | 2 | 0 | 2 | 4 |
| Lee | One Year Experience of the Hand Allotransplantation First Performed after Korea Organ Transplantation Act Amendment | DOI: 10.1055/a-2059-5570. | 2 | 0 | 2 | 4 |
| Ozmen | First Composite Woman-to-Woman Facial Transplantation in Turkey: Challenges and Lessons to Be Learned | DOI: 10.1097/SAP.0000000000003323 | 2 | 0 | 2 | 4 |
| Murakami | Low-dose interleukin-2 promotes immune regulation in face transplantation: A pilot study | DOI: 10.1016/j.ajt.2023.01.016 | 2 | 1 | 2 | 5 |
| Zaccardelli | Case Report: Post-transplant lymphoproliferative disorder as a serious complication of vascularized composite allotransplantation | DOI: 10.3389/frtra.2024.1339898 | 2 | 0 | 2 | 4 |
